# #Nitrosocarbonyls 1: Antiviral Activity of *N*-(4-Hydroxycyclohex-2-en-1-yl)quinoline-2-carboxamide against the Influenza A Virus H1N1

**DOI:** 10.1155/2014/472373

**Published:** 2014-12-31

**Authors:** Dalya Al-Saad, Misal Giuseppe Memeo, Paolo Quadrelli

**Affiliations:** Department of Chemistry, University of Pavia, Viale Taramelli 12, 27100 Pavia, Italy

## Abstract

Influenza virus flu A H1N1 still remains a target for its inhibition with small molecules. Fleeting nitrosocarbonyl intermediates are at work in a short-cut synthesis of carbocyclic nucleoside analogues. The strategy of the synthetic approaches is presented along with the *in vitro* antiviral tests. The nucleoside derivatives were tested for their inhibitory activity against a variety of viruses. Promising antiviral activities were found for specific compounds in the case of flu A H1N1.

## 1. Introduction

Influenza virus flu A H1N1 still remains a target for its inhibition with small molecules. The outbreak of the human pandemic influenza A (H1N1) caused a considerable public concern. Recent studies focused the attention on the understanding of novel viruses by analyzing the relationship between molecular characteristics and pathogenic properties [1]. Results of this analysis indicated that the human pandemic influenza A (H1N1) virus was a new reassorted virus combining genetic materials from the avian flu (H1N1) virus, classical swine flu (H1N1) virus, human flu (H3N2) virus, and Eurasian swine flu (H1N1) virus. The analysis of the sequences for receptor-binding and cleavage sites of hemagglutinin (HA) allowed for determining that replication could be inhibited by molecules such as oseltamivir (Tamiflu), a diamine derivative of the shikimic acid [2], and zanamivir (Relenza), a derivative of the 3,4-diamino-2-(hydroxymethyl)-3,4-dihydro-2*H*-pyran-6-carboxylic acid [3–5].

The influenza A virus is an orthomyxovirus, constituted by receptor-binding complexes containing two primary structural proteins: hemagglutinin (HA) and neuraminidase (NA). HA is the primary protein responsible for binding to receptor sites on the cell membrane, allowing the virion to enter the cell [4–8].

On pursuing our studies and in the search of novel small molecules with potential antiviral activity, we prepared some nucleoside derivatives, found moderately active against human herpes and varicella viruses [9] or strongly active against the human papilloma virus (HPV) [10] as well as influenza A virus H1N1 [11]. The synthetic protocol relied upon the nitrosocarbonyl chemistry.

Nitrosocarbonyls are fleeting intermediates whose generation as well as synthetic applications represents a remarkable piece of organic chemistry [12]. The nitrosocarbonyl intermediates** 1** (R-CONO) are highly reactive species discovered at the beginning of the seventies by Kirby and their powerful dienophilic and enophilic activities were reported in 1973 in his seminal paper [13].

**Scheme 1:**
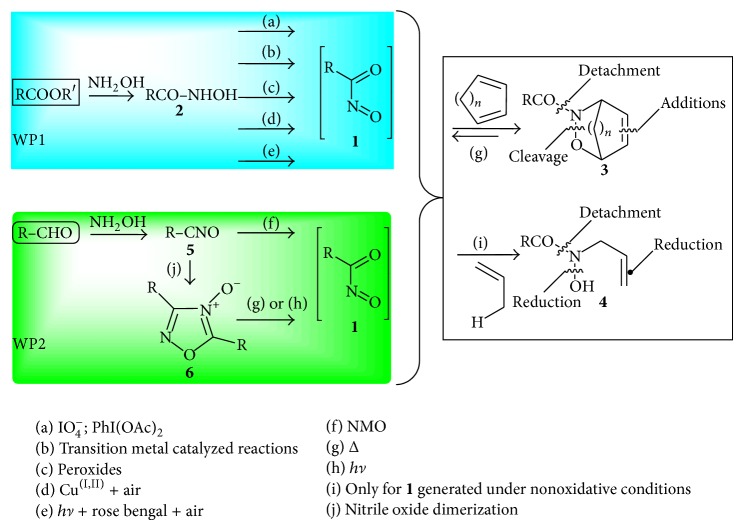
Generation methods of nitrosocarbonyl intermediates and reactivity with dienes and alkenes.

Nitrosocarbonyl or acylnitroso intermediates attracted a great deal of attention in the area during the last decades since highly functionalized molecules can be readily achieved [13–16]. The generation of nitrosocarbonyls** 1** can be easily performed according to two main synthetic approaches summarized in the two working packages (WP1 and WP2) reported in Scheme 1.

WP1 shows the use of hydroxamic acids** 2** as starting compounds whose oxidation is performed either with the more soluble tetraalkylammonium periodate salts or PhI(OAc)_2_ (path a) [17]. Other oxidative conditions were developed such as the use of transition metal catalyzed reactions [18–21] and peroxides [22, 23] (paths b and c). Recently, the oxidation with copper salts in the presence of air [24, 25] (path d) or photochemical sensibilized oxidation processes [26] (path e) became valuable mild methods for nitrosocarbonyl generation [27].

WP2 shows the use of nitrile oxides** 5** for the case at hand; these 1,3-dipoles find in the wide family of the aldehydes the main and most convenient source with the possibility of proposing a variety of substituents. This valuable alternative towards nitrosocarbonyls** 1** relies upon the mild oxidation of nitrile oxides** 5** with* N*-methylmorpholine-*N*-oxide (path f, NMO) [28] and the clean thermolysis (path g) and photolysis (path h) of 1,2,4-oxadiazole-4-oxides** 6**, the most reactive dimer of nitrile oxides** 5** [29, 30].

**Scheme 2:**
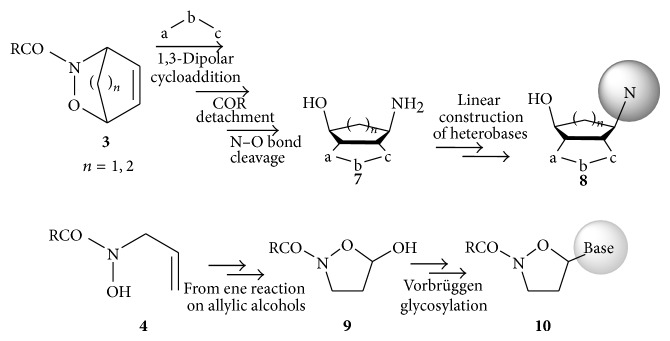
Synthesis of nucleoside analogues from HDA and ene adducts of nitrosocarbonyl intermediates.

During our studies, we developed the synthesis of a new class of isoxazoline-carbocyclic nucleosides** 8** starting from the regioisomeric aminols** 7** (Scheme 2) through elaboration of the HDA cycloadducts** 3** [31–34]. From the ene adduct of nitrosocarbonyls and the 3-methyl-2-buten-1-ol, the isoxazolidine** 9**, the preparation of new* N,O*-nucleoside pyrimidine analogues** 10** was achieved through the Vorbrüggen protocol [35, 36]. We wish to report here the application of the WP2 protocol for the rapid and easy synthesis of new promising active nucleoside analogues. This simplified synthetic strategy implies the replacement of traditional heterobases, such as purines or pyrimidines, with selected and easy available heterocycles containing one nitrogen atom; once prepared, the products are submitted for* in vitro* tests to determine eventual activity and as a consequence further structural modifications. The required heterocyclic aldehydes containing one nitrogen atom are converted into the corresponding nitrile oxides as convenient source for the required nitrosocarbonyl intermediates.

## 2. Results

### 2.1. Oxidation of Nitrile Oxides and Cleavage of N–O Bonds

**Scheme 3:**
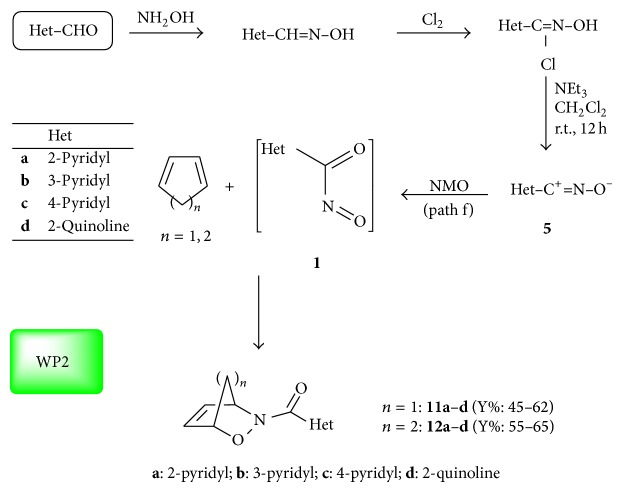
Application of the WP2 protocol to the synthesis of nitrosocarbonyl HDA cycloadducts** 11a**–**d** and** 12a**–**d** from heteroaromatic nitrile oxides.

Pyridine and quinoline hydroximoyl chlorides were prepared according to the literature procedures (Scheme 3) [37–39]. The selected aldehydes were converted in high yields into the corresponding oximes following the classical methods [37–39] and from the latter the desired hydroximoyl chlorides were obtained upon chlorination with chlorine gas in chloroform as solvent at −20°C for 1.5 h. The* in situ* generation of the nitrile oxides** 5a**–**d** (Et_3_N, 1.2 equiv.) is required in the present cases since none of the nitrile oxides at hand displayed any stability at room temperature, possibly in the solid state, for a long time. The mild oxidation of these 1,3-dipoles with* N*-methyl-morpholine* N*-oxide (NMO, 1.3 equiv.) is conducted one-pot in the presence of the required trapping diene (freshly distilled cyclopentadiene or 1,3-cyclohexadiene, 2 equiv.) to afford the nitrosocarbonyl HDA cycloadducts** 11a**–**d** and** 12a**–**d**.

Compounds** 11a**–**d** and** 12a**–**d** were isolated from good to high yields (45–65%) and fully characterized from their analytical and spectroscopic data, except for** 11d**, already reported [11]. For the new products, the spectroscopic data are consistent with the reported structures; we wish to point out the most relevant and diagnostic ^1^H NMR (CDCl_3_) signals relative to the 2,3-oxazanorborn-5-ene moieties in the products** 11** at hand. The olefinic protons and the deshielded bridge-head protons were found in the range *δ* = 6.37–6.67 and *δ* = 5.37–6.16, respectively. Similarly, the 2,3-oxazabicyclo[2.2.2]oct-5-ene moieties of the products** 12** are clearly observed in the ^1^H NMR (CDCl_3_) spectra. The olefinic protons and the deshielded bridge-head protons were found, respectively, in the ranges *δ* = 5.74–6.71 and *δ* = 4.79–5.55.

**Scheme 4:**
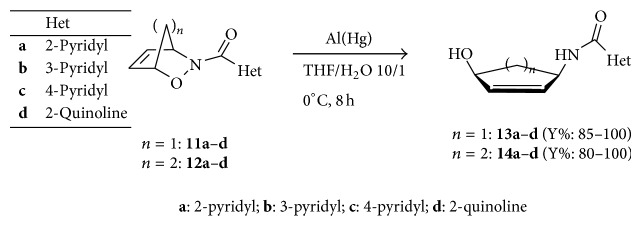
Reductive cleavage of N–O bonds and synthesis of the 4-hydroxycyclopent-2-enyl heterocyclic amides** 13a**–**d** and** 14a**–**d**.

Compounds** 11a**–**d** and** 12a**–**d** were then submitted to the mild reductive cleavage of the N–O bond using the Al(Hg) amalgam in THF/H_2_O as solvents at 0°C [40]. The 4-hydroxycyclopent-2-enyl heterocyclic amides** 13a**–**d** and** 14a**–**d** were obtained in very high yields and the structures were attributed on the basis of their fully consistent analytical and spectroscopic data (Scheme 4).

The most relevant and diagnostic ^1^H NMR signals are those relative to the olefinic protons of the cyclopentene and cyclohexene moieties found at *δ* = 5.94–6.15 (dd, *J* = 5 Hz in CDCl_3_) and at *δ* = 5.66–6.05 (dd, *J* = 10 Hz in DMSO), respectively. All the other signals are found in the expected range for the given attributions.

Samples of the compounds** 13a**–**d** and** 14a**–**d** were straight submitted for a primary* in vitro* antiviral screening in collaboration with the NIH/NIAID (USA).

### 2.2. Antiviral Activity

This rapid access to the target molecules allowed for their preparation in good amounts suitable for several biological tests. Compounds** 13a**–**d** and** 14a**–**d** were tested for their inhibitory activity against herpes simplex virus 1 (HSV-1), herpes simplex virus 2 (HSV-2), vaccinia virus (VV), and hepatitis B virus (HBV). The antiviral activity of the above-reported compounds was tested* in vitro* in cell line HFF (strain E-377 for HSV-1, strain MS for HSV-2, and strain Copenhagen for VV) and cell line HepG 2.2.2.15 (strain ayw1) for HBV. A test against human papilloma virus (HPV) was conducted on HEK 293 cells (strain HPV-11). Finally, the compounds were tested against respiratory virus influenza A H1N1 cell line MDCK (strain California 07/2009) and neuraminidase (NA) cell line HFF (strain NA).

Both compounds** 13a**–**d** and** 14a**–**d** were found inactive against HSV-1 and -2; EC_50_ and CC_50_ values were >300 with a SI_50_ = 1 (drug concentration range 0.096–300 *μ*M; acyclovir control concentration range 0.032–100 *μ*M). Compounds** 13a**–**d** and** 14a**–**d** also displayed a complete inactivity against the VV; EC_50_ and CC_50_ values were >300 with a SI_50_ = 1 (drug concentration range 0.096–300 *μ*M; cidofovir control concentration range 0.096–300 *μ*M).

The* in vitro* tests against HBV revealed a substantial inactivity of compounds** 13a**–**d** and of compounds** 14a**,** b**, and** d** since the EC_50_ and CC_50_ values were >20 with a SI_50_ = 1 (drug concentration range 0.063–20 *μ*M; 3TC control concentration range 0.00032–0.1 *μ*M). Compound** 14c** only gave a different value of EC_50_ = 9.64 even though the CC_50_ value was found >100 with a SI_50_ = 1, while the cidofovir used as reference data gave EC_50_ = 180, CC_50_ > 200 with a SI_50_ > 1.

The* in vitro* tests against HPV revealed a complete inactivity of compounds** 13a**–**d** and** 14a**–**d** since the EC_50_ and CC_50_ values were >20 with a SI_50_ = 1 (drug concentration range 0.063–20 *μ*M; 3TC control concentration range 0.00032–0.1 *μ*M). The NA tests gave for compounds** 13b**,** c** and** 14a**–**c** comparable results (CC_50_ > 300) with those of cidofovir used as control drug (drug assay: neutral red (toxicity); drug concentration range 0.096–300 mM; cidofovir control concentration range 0.096–300 mM).

The derivatives** 13a–d** and** 14a–d** were also tested against the respiratory influenza A H1N1 virus and Table 1 reports the primary antiviral activities of the tested compounds and control experiments on the specified drug. The majority of the compounds were found to be inactive, but in a single case** 14d** (Table 1, entries 15 and 16) the EC_50_ values are just 10 times those of the reference compound while the SI_50_ values require to be increased by the same factor.

## 3. Discussion and Conclusions

These primary antiviral activity data represent somewhat the prelude to possible developments for more active compounds through modifications of the synthetic pathway, chosen on the basis of previous results [11]. Indeed the synthesis is suitable for convenient, easy, and straightforward structural modifications. The larger activity shown by those compounds, which bear a naphthyl-like residue, that is, the quinolone ring, could suggest a possible mechanism based on DNA intercalation. Polycyclic aromatic hydrocarbons are known as DNA-intercalating agents and molecular modeling studies on these structures confirm the degree of binding when a polycyclic aromatic residue is linked to an heterocyclic ring [41, 42].

Polyaromatic groups may be active through their ability to establish *π*-*π* stacking interactions with themselves as well as DNA intercalators; these mechanisms are in action in different cases depending upon the biological targets [43].

**Figure 1:**
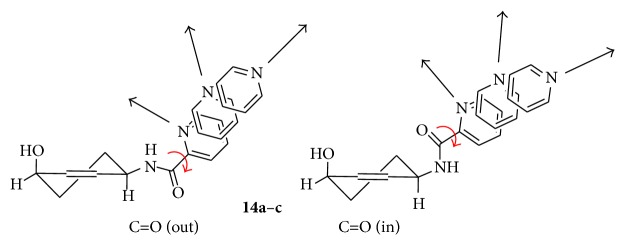
Orientations of the pyridine rings in conformers** 14a–c**. Red arrows indicate rotation around the C=O-Het bond for all the possible conformers of the pyridine rings.

An heterocyclic ring bearing a single heteroatom (the pyridine ring) seems to be somewhat inefficient from the biological point of view even though the nitrogen atom orientation can be easily changed in compound** 14** from** a** to** c** (Figure 1).

**Figure 2:**
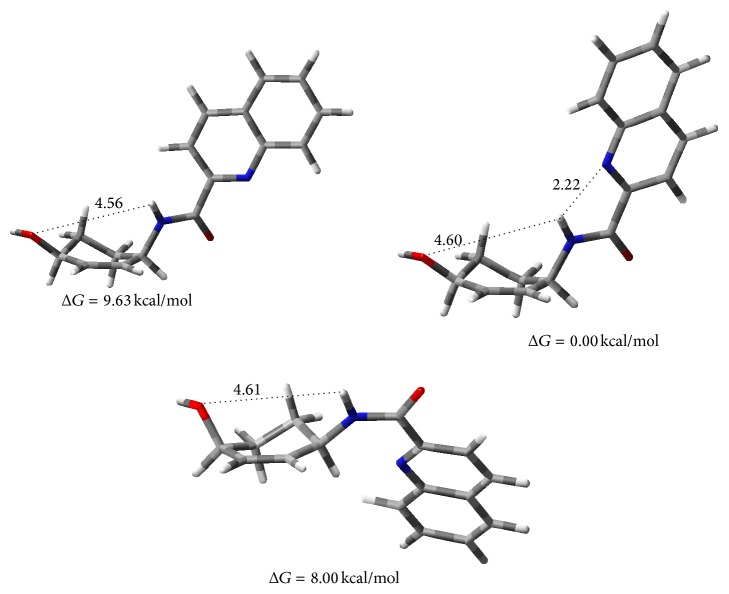
Conformers of compound** 14d**.

On the other hand, the quinoline ring possesses the structural features to display this type of action mechanism as shown in many cases in the recent literature [44, 45]. The quinoline is a naphthalene-like heterocycle and assumes different orientations suitable to adapt the structure during the interaction with DNA or active sites of proteins involved into the virus replication (DFT conformational studies at the B3LYP/6-31G(d) [46] level; Figure 2).

Docking studies are pursued to determine the key point concerning the SAR able to orientate positively the synthesis towards a specific target. Moreover, the fragment-based drug design (FBDD) represents a challenging approach in the case at hand; the reported structures seem to fit nicely with the application of computational FBDD for selecting the structural modification to increase the biological activity [47, 48].

The simplified syntheses described rely upon the valuable chemistry of nitrosocarbonyl intermediates and the easy synthetic elaboration of the corresponding hetero-Diels-Alder cycloadducts. The replacement of traditional heterobases, such as purines or pyrimidines, with selected and easy available heterocycles containing one nitrogen atom, although simple or elemental, allows for a viral target oriented production of small molecules able to display some inhibition activity and suitable for further development under structural modification suggested by docking as well as FBDD analyses.

## 4. Experimental Section

### 4.1. General

All melting points are uncorrected. Elemental analyses were done on a C. Erba 1106 elemental analyzer. IR spectra (Nujol mulls) were recorded on an FT-IR Perkin-Elmer RX-1. ^1^H- and ^13^C-NMR spectra were recorded on a Bruker AVANCE 300 in the specified deuterated solvents. Chemical shifts are expressed in ppm from internal tetramethylsilane (*δ*). Column chromatography and tlc: silica gel 60 (0.063–0.200 mm) (Merck); eluant cyclohexane/ethyl acetate from 9 : 1 to 5 : 5. The identification of samples from different experiments was secured by mixed mps and superimposable IR spectra. Compound purification: all the products described were suitably purified; solid compounds were recrystallized from proper solvents and oily compounds were purified through bulb-to-bulb distillation (Kugelrohr distillation, Buchi GKR-51).

### 4.2. Materials

Cyclopentadiene was freshly distilled from the dimer purchased from Sigma-Aldrich. 1,3-Cyclohexadiene, pyridine-carbaldehyde, 2-quinoline-carbaldehyde, and 4-bromothiazole-2-carbaldehyde were purchased from Sigma-Aldrich. All other reagents and solvents were purchased from Sigma-Aldrich and Alfa-Aesar and used without any further purification. The heteroaromatic hydroximoyl chlorides required for the* in situ* generation of the nitrile oxides** 5** were prepared according to the literature reported procedures [26].

### 4.3. Synthesis of Cycloadducts **11a**–**d** and **12a**–**d**

To a dichloromethane (DCM, 50 mL) solution of 1.5 equivalents of NMO and 2.1 equivalents of Et_3_N, 2 equivalents of freshly distilled cyclopentadiene were added. The mixture was then cooled down with ice and a DCM (50 mL) solution containing the heteroaromatic hydroximoyl chloride of choice (1 equiv.) was added dropwise under vigorous stirring at 0°C for a couple of hours. The reaction was left under stirring at room temperature for 48 h. After this period of time, the reaction is quenched by pouring the mixture in brine (100 mL) and the products were extracted from the water phase with DCM (2 × 50 mL). The residue obtained upon evaporation of the solvent was crystallized from proper solvent. Compounds** 11a–d** and** 12a–d** were obtained in the reported yield and fully characterized.

### 4.4. *N*-Pyridin-2-oyl 2,3-Oxazanorborn-5-ene **11a**

1.24 g (59%), oil. IR: *ν* = 1734 (C=O), 1655 (C=N) cm^−1^. ^1^H NMR (300 MHz, CDCl_3_, 25°C): *δ* = 1.87 and 2.17 (d, 1H + 1H, *J* = 9 Hz, CH_2_), 5.41 (s, 1H, CH–N), 6.16 (s, 1H, CH–O), 6.37 (s, 2H, CH=CH), 7.40, 7.80, 7.99 and 8.63 (m, 4H, 2-Py). ^13^C NMR (75 MHz, CDCl_3_, 25°C): *δ* = 47.9; 66.3; 83.3; 124.0; 125.3; 132.0; 133.5; 136.5; 147.8; 151.1; 164.7. C_11_H_10_O_2_N_2_ (202.21): calcd. C, 65.34; H, 4.98; N, 13.85; found C 65.30, H 4.92, N 13.84.

### 4.5. *N*-Pyridin-3-oyl 2,3-Oxazanorborn-5-ene **11b**

1.30 g (62%), oil. IR: *ν* = 1701 (C=O), 1648 (C=N) cm^−1^. ^1^H NMR (300 MHz, CDCl_3_, 25°C): *δ* = 1.91 and 2.15 (d, 1H + 1H, *J* = 9 Hz, CH_2_), 5.37 (s, 2H, CH–N and CH–O), 6.40 and 6.64 (s, 2H, CH=CH), 7.37, 8.12, 8.70 and 9.02 (m, 4H, 3-Py). ^13^C NMR (75 MHz, CDCl_3_, 25°C): *δ* = 48.3; 63.5; 85.0; 123.0; 129.9; 133.2; 136.8; 149.6; 151.6; 169.3; 174.2. C_11_H_10_O_2_N_2_ (202.21): calcd. C, 65.34; H, 4.98; N, 13.85; found C 65.35, H 4.91, N 13.83.

### 4.6. *N*-Pyridin-4-oyl 2,3-Oxazanorborn-5-ene **11c**

1.07 g (51%), oil. IR: *ν* = 1705 (C=O), 1652 (C=N) cm^−1^. ^1^H NMR (300 MHz, CDCl_3_, 25°C): *δ* = 2.01 and 2.19 (d, 1H + 1H, *J* = 9 Hz, CH_2_), 5.45 (s, 1H, CH–N), 5.58 (bs, 1H, CH–O), 6.46 and 6.75 (s, 2H, CH=CH), 7.98 and 8.78 (m, 4H, 4-Py). ^13^C NMR (75 MHz, CDCl_3_, 25°C): *δ* = 48.5; 65.1; 85.6; 124.4; 130.2; 132.4; 133.5; 136.7; 145.3; 153.3; 160.2. C_11_H_10_O_2_N_2_ (202.21): calcd. C, 65.34; H, 4.98; N, 13.85; found C 65.30, H 4.92, N 13.82.

### 4.7. *N*-Pyridin-2-oyl 2,3-Oxazabicyclo[2.2.2]oct-5-ene **12a**

1.34 g (60%), m.p. 110–113°C from ethanol. IR: *ν* = 1637 (C=O and C=N) cm^−1^. ^1^H NMR (300 MHz, CDCl_3_, 25°C): *δ* = 1.55 and 2.35 (d, 2H + 2H, *J* = 8 Hz, CH_2_CH_2_), 4.97 (bs, 1H, CH–N), 5.55 (s, 1H, CH–O), 6.59 (s, 2H, CH=CH), 7.35, 7.76, 7.88 and 8.62 (m, 4H, 2-Py). ^13^C NMR (75 MHz, CDCl_3_, 25°C): *δ* = 21.7, 23.3; 51.5; 71.8; 124.8; 130.9; 131.8; 136.8; 148.5; 152.6; 162.2. C_12_H_12_O_2_N_2_ (216.24): calcd. C, 66.65; H, 5.59; N, 12.96; found C 66.60, H 5.52, N 12.94.

### 4.8. *N*-Pyridin-3-oyl 2,3-Oxazabicyclo[2.2.2]oct-5-ene **12b**

1.46 g (65%), m.p. 81-82°C from ethanol. IR: *ν* = 1641 (C=O and C=N) cm^−1^. ^1^H NMR (300 MHz, CDCl_3_, 25°C): *δ* = 1.56 and 2.23 (m, 2H + 2H, CH_2_CH_2_), 4.79 (bs, 1H, CH–N), 5.45 (b, 1H, CH–O), 6.54 and 6.71 (m, 2H, CH=CH), 7.31, 8.00, 8.63 and 8.91 (m, 4H, 3-Py). ^13^C NMR (75 MHz, CDCl_3_, 25°C): *δ* = 20.8; 23.3; 46.8; 72.1; 122.7; 129.8; 131.6; 133.1; 136.4; 149.1; 151.2; 166.0. C_12_H_12_O_2_N_2_ (216.24): calcd. C, 66.65; H, 5.59; N, 12.96; found C 66.61, H 5.53, N 12.97.

### 4.9. *N*-Pyridin-4-oyl 2,3-Oxazabicyclo[2.2.2]oct-5-ene **12c**

1.23 g (55%), m.p. 83–85°C from ethanol. IR: *ν* = 1645 (C=O and C=N) cm^−1^. ^1^H NMR (300 MHz, CDCl_3_, 25°C): *δ* = 1.54 and 2.23 (m, 2H + 2H, CH_2_CH_2_), 4.78 (bs, 1H, CH–N), 5.45 (bs, 1H, CH–O), 6.56 and 6.74 (m, 2H, CH=CH), 7.53 and 8.67 (m, 4H, 4-Py). ^13^C NMR (75 MHz, CDCl_3_, 25°C): *δ* = 20.7; 23.3; 46.8; 72.2; 122.6; 131.6; 133.1; 141.3; 149.5; 150.2; 165.6. C_12_H_12_O_2_N_2_ (216.24): calcd. C, 66.65; H, 5.59; N, 12.96; found C 66.62, H 5.58, N 12.96.

### 4.10. *N*-Quinol-2-oyl 2,3-Oxazabicyclo[2.2.2]oct-5-ene **12d**

1.60 g (58%), m.p. 148–150°C from ethanol. IR: *ν* = 1652 (C=O and C=N) cm^−1^. ^1^H NMR (300 MHz, CDCl_3_, 25°C): *δ* = 1.60 and 2.47 (d, 2H + 2H, *J* = 8 Hz, CH_2_CH_2_), 5.05 (s, 1H, CH–N), 5.74 (s, 1H, CH–O), 6.62 (s, 2H, CH=CH), 7.63, 7.81, 7.87, 7.99, 8.13 and 8.28 (m, 6H, 2-Qui). ^13^C NMR (75 MHz, CDCl_3_, 25°C): *δ* = 21.9, 23.3; 51.7; 71.8; 121.4; 127.6; 127.7; 129.8; 129.9; 131.6; 132.0; 137.0; 146.3; 151.9; 161.9. C_16_H_14_O_2_N_2_ (266.29): calcd. C, 72.16; H, 5.30; N, 10.52; found C 72.12, H 5.32, N 10.54.

### 4.11. Reductive Cleavage of N–O Bonds and Synthesis of Compounds **13a**–**d** and **14a**–**d**

To a stirred solution of the cycloadducts** 11a**–**d** and** 12a**–**d** (1 g) in 200 mL of THF/H_2_O 10/1 under nitrogen and at 0°C, 1 g of Al(Hg) is added portionwise and the reaction is conducted until complete consumption of the starting materials (monitored by TLC). After completion, the solutions were diluted with THF and filtered over celite. The organic phases were then evaporated to dryness to leave the corresponding aminols** 13a**–**d** and** 14a**–**d** in the reported yield and were fully characterized.

### 4.12. *N*-(4-Hydroxycyclopent-2-enyl)picolinamide **13a**

0.99 g (98%), oil. IR: *ν* = 3370 (OH), 1655 (C=O) cm^−1^. ^1^H NMR (300 MHz, CDCl_3_, 25°C): *δ* = 1.79 (dt, 1H, *J* = 14, 3.5 Hz, HC-*H*), 1.82 (b, 1H, OH), 2.88 (dt, 1H, *J* = 14, 7.5 Hz,* H*-CH), 4.86 (m, 2H, CH–N and CH–O), 5.94 and 6.13 (m, 1H + 1H, CH=CH), 7.45, 7.87, 8.20 and 8.56 (m, 4H, 2-Py), 8.55 (bs, 1H, NH). ^13^C NMR (75 MHz, CDCl_3_, 25°C): *δ* = 41.1; 54.0; 75.5; 122.1; 126.2; 133.3; 136.8; 137.4; 147.9; 149.7; 163.7. C_11_H_12_O_2_N_2_ (204.23): calcd. C, 64.69; H, 5.92; N, 13.72; found C 64.70, H 5.92, N 13.74.

### 4.13. *N*-(4-Hydroxycyclopent-2-enyl)nicotinamide **13b**

0.98 g (97%), m.p. 125–127°C from ethyl acetate. IR: *ν* = 3350 (OH), 1652 (C=O) cm^−1^. ^1^H NMR (300 MHz, CDCl_3_, 25°C): *δ* = 1.73 (dt, 1H, *J* = 14, 3 Hz, HC-*H*), 2.85 (dt, 1H, *J* = 14, 8 Hz,* H*-CH), 3.30 (b, 2H, OH e NH), 4.83 (d, 1H, *J* = 7 Hz, CH–O), 5.07 (m, 1H, CH–N), 5.96 and 6.09 (d, 1H + 1H, *J* = 4 Hz, CH=CH), 7.48, 8.27, 8.70 and 9.17 (m, 4H, 3-Py). ^13^C NMR (75 MHz, CDCl_3_, 25°C): *δ* = 40.8; 53.9; 75.2; 123.8; 130.6; 133.7; 136.5; 136.7; 147.3; 151.0; 164.4. C_11_H_12_O_2_N_2_ (204.23): calcd. C, 64.69; H, 5.92; N, 13.72; found C 64.71, H 5.93, N 13.75.

### 4.14. *N*-(4-Hydroxycyclopent-2-enyl)isonicotinamide **13c**

1.00 g (100%), m.p. 170–175°C from ethyl acetate. IR: *ν* = 3326 (OH), 1640 (C=O) cm^−1^. ^1^H NMR (300 MHz, CDCl_3_, 25°C): *δ* = 1.71 (dt, 1H, *J* = 14, 3 Hz, HC-*H*), 2.34 (s, 1H, OH), 2.85 (dt, 1H, *J* = 14, 8 Hz,* H*-CH), 4.84 (d, 1H, *J* = 7 Hz, CH–O), 5.00 (m, 1H, CH–N), 5.97 and 6.13 (m, 1H + 1H, *J* = 5 Hz, CH=CH), 6.64 (d, 1H, *J* = 7 Hz, NH), 7.66 and 8.76 (m, 4H, 4-Py). ^13^C NMR (75 MHz, CDCl_3_, 25°C): *δ* = 41.1; 54.3; 75.2; 120.9; 133.4; 137.1; 141.6; 150.2; 164.7. C_11_H_12_O_2_N_2_ (204.23): calcd. C, 64.69; H, 5.92; N, 13.72; found C 64.71, H 5.91, N 13.73.

### 4.15. *N*-(4-Hydroxycyclopent-2-enyl)quinoline-2-carboxamide **13d**

0.86 g (85%), m.p. 89-90°C from ethyl acetate. IR: *ν* = 3420 (OH), 3357 (NH), 1667 (C=O) cm^−1^. ^1^H NMR (300 MHz, CDCl_3_, 25°C): *δ* = 1.85 (dt, 1H, *J* = 14, 4 Hz, HC-*H*), 2.86 (b, 1H, OH), 2.92 (dt, 1H, *J* = 14, 8 Hz,* H*-CH), 4.84 (m, 1H, CH–O), 4.95 (m, 1H, CH–N), 5.98 and 6.15 (m, 1H + 1H, CH=CH), 7.63, 7.77, 7.86, 8.09 and 8.29 (m, 6H, 2-Qui), 8.62 (d, 1H, *J* = 7 Hz, NH). ^13^C NMR (75 MHz, CDCl_3_, 25°C): *δ* = 41.0; 54.1; 75.4; 118.7; 127.6; 127.9; 129.2; 129.4; 130.2; 133.3; 136.9; 137.6; 146.2; 149.4; 163.9. C_15_H_14_N_2_O_2_ (254.28): calcd. C, 70.85; H, 5.55; N, 11.02; found C 70.81, H 5.51, N 11.03.

### 4.16. *N*-(4-Hydroxycyclohex-2-enyl)picolinamide **14a**

0.95 g (94%), oil. IR: *ν* = 3378 (OH), 1667 (C=O) cm^−1^. ^1^H NMR (300 MHz, DMSO, 25°C): *δ* = 1.62 and 1.77 (m, 4H, CH_2_CH_2_), 4.00 (s, 1H, CH–O), 4.43 (m, 1H, CH–N), 4.78 (d, 1H, *J* = 6 Hz, OH), 5.67 and 5.86 (m, 1H + 1H, CH=CH), 7.63, 8.04 and 8.64 (m, 4H, 2-Py), 8.33 (d, 1H, *J* = 5 Hz, NH). ^13^C NMR (75 MHz, DMSO, 25°C): *δ* = 25.1; 28.8; 44.1; 63.0; 121.8; 126.7; 128.7; 134.1; 137.9; 148.5; 149.6; 162.9. C_12_H_14_O_2_N_2_ (218.25): calcd. C, 66.04; H, 6.47; N, 12.84; found C 66.01, H 6.42, N 12.84.

### 4.17. *N*-(4-Hydroxycyclohex-2-enyl)nicotinamide **14b**

0.93 g (92%), m.p. 95–99°C from ethyl acetate. IR: *ν* = 3286 (OH), 1661 (C=O) cm^−1^. ^1^H NMR (300 MHz, DMSO, 25°C): *δ* = 1.72 (m, 4H, CH_2_CH_2_), 4.03 (s, 1H, CH–O), 4.43 (m, 1H, CH–N), 5.66 and 5.82 (d, 1H + 1H, *J* = 9 Hz, CH=CH), 7.51, 8.25, 8.70 and 9.03 (m, 4H, 3-Py), 8.74 (d, 1H, *J* = 8 Hz, NH). ^13^C NMR (75 MHz, DMSO, 25°C): *δ* = 24.8; 28.9; 45.1; 62.7; 123.5; 129.3; 130.0; 133.3; 135.6; 148.3; 151.4; 164.3. C_12_H_14_O_2_N_2_ (218.25): calcd. C, 66.04; H, 6.47; N, 12.84; found C 66.03, H 6.43, N 12.86.

### 4.18. *N*-(4-Hydroxycyclohex-2-enyl)isonicotinamide **14c**

1.00 g (100%), m.p. 245–250°C (dec.) from ethyl acetate. IR: *ν* = 3306 (OH), 1651 (C=O) cm^−1^. ^1^H NMR (300 MHz, DMSO, 25°C): *δ* = 1.71 (m, 4H, CH_2_CH_2_), 4.15 (s, 1H, CH–O), 4.41 (m, 1H, CH–N), 5.63 and 5.83 (d, 1H + 1H, *J* = 9 Hz, CH=CH), 7.79 and 8.70 (m, 4H, 4-Py), 8.81 (d, 1H, *J* = 8 Hz, NH). ^13^C NMR (75 MHz, DMSO, 25°C): *δ* = 24.7; 28.9; 45.2; 62.7; 121.5; 124.9; 129.1; 133.3; 141.4; 150.0; 164.3. C_12_H_14_O_2_N_2_ (218.25): calcd. C, 66.04; H, 6.47; N, 12.84; found C 66.06, H 6.41, N 12.85.

### 4.19. *N*-(4-Hydroxycyclohex-2-enyl)quinoline-2-carboxamide **14d**

0.81 g (80%), m.p. 80–84°C from ethyl acetate. IR: *ν* = 3382 (OH), 1668 (C=O) cm^−1^. ^1^H NMR (300 MHz, CDCl_3_, 25°C): *δ* = 1.99 (m, 4H, CH_2_CH_2_), 4.31 (s, 1H, CH–O), 4.72 (m, 1H, CH–N), 5.92 and 6.06 (d, 1H + 1H, *J* = 9 Hz, CH=CH), 7.66, 7.77, 7.79, 8.13 and 8.33 (m, 6H, 2-Qui), 8.23 (d, 1H, *J* = 8 Hz, NH). ^13^C NMR (75 MHz, CDCl_3_, 25°C): *δ* = 25.3; 29.1; 44.8; 64.5; 118.7; 127.6; 127.9; 129.2; 129.5; 130.1; 130.7; 132.6; 137.5; 146.3; 149.5; 163.7. C_16_H_16_O_2_N_2_ (268.31): calcd. C, 71.62; H, 6.01; N, 10.44; found C 71.66, H 6.01, N 10.45.

### 4.20. Computational Methods

All calculations were carried out using the Gaussian 09 [46] program package through optimizations in the gas phase at the B3LYP/6-31G(d) level. Vibrational frequencies were computed to verify that the optimized structures were minima.

### 4.21. Antiviral Assays

The National Institute of Allergy and Infectious Diseases (NIAID) established the AACF under a contract with Southern Research Institute. The NIAID, through the AACF, provides free and confidential services for suppliers, who are interested in submitting compounds to be evaluated for antiviral activity. Tested compounds were delivered in standard DMSO solutions. The methods applied for the different assays can be found at the URL via the internet at http://niaid-aacf.org/.

The antiviral tests were conducted on the derivatives** 13a**–**d** and** 14a**–**d** suitably purified; compounds** 13b**–**d** and** 14b**–**d** were recrystallized from proper solvents and compounds** 13a** and** 14a** were purified through bulb-to-bulb distillation (Kugelrohr distillation).

The following control assays were applied: herpes simplex virus 1 (crystal violet), herpes simplex virus 2 (crystal violet), vaccinia virus (crystal violet), hepatitis B virus (DNA hybridization/Luciferase reporter/CytoTox-1). The antiviral activity of the above-reported compounds was tested* in vitro* in cell line HFF (strain E-377 for HSV-1, strain G for HSV-2, strain Ellen for VZV, and strain Copenhagen for VV), Vero 76 cell line (strain Adames) for PTV, cell line 2.2.15 (strain ayw) for HBV, and cwell line Huh-Luc/Neo ET (strain CON-1) for HCV. The following control assays were applied: influenza A H1N1 cell line MDCK (strain California 7/2009; CellTiter-Glo) and neuraminidase (NA) cell line HFF (strain NA; neutral Red). HPV (HEK 293 cells, strain HPV-11).

Control drug reference data: HSV-1: acyclovir, EC_50_ 3.29; CC_50_ > 100, SI_50_ > 30; control assay: crystal violet (cytopathic effect/toxicity). HSV-2: acyclovir, EC_50_ 3.7; CC_50_ > 100, SI_50_ > 27; control assay: crystal violet (cytopathic effect/toxicity). VV: cidofovir, EC_50_ 6.07; CC_50_ > 300, SI_50_ > 4.9; control assay: crystal violet (cytopathic effect/toxicity). HBV: 3TC, EC_50_ 0.01; CC_50_ > 0.1, SI_50_ > 10; control assay: polymerase chain reaction (Virion/CellTiter 96, toxicity). H1N1: ribavirin, EC_50_ 3.2; CC_50_ > 100, SI_50_ > 31; control assay: visual and neutral red. NA: cidofovir, CC_50_ > 300; control assay: neutral red. HSV-1: cidofovir, EC_50_ 180; CC_50_ > 200, SI_50_ > 1; control assay: crystal violet polymerase chain reaction (DNA/cell proliferation, toxicity).

## Supplementary Material

1. 1H and 13C NMR spectra.
2. Cartesian coordinates of calculated structures.

## Figures and Tables

**Table 1:** Primary antiviral activities of compounds **13a–d** and **14a–d** against influenza A H1N1 virus.

Entry	Compounds	EC_50_	CC_50_^[c]^	SI_50_
1	13**a** ^[a]^	>100	>100	0
2	13**a** ^[b]^	>100	>100	0
3	13**b** ^[a]^	>100	>100	0
4	13**b** ^[b]^	>100	>100	0
5	13**c** ^[a]^	>100	>100	0
6	13**c** ^[b]^	>100	>100	0
7	13**d** ^[a]^	>100	>100	0
8	13**d** ^[b]^	>100	>100	0
9	14**a** ^[a]^	>100	>100	0
10	14**a** ^[b]^	>100	>100	0
11	14**b** ^[a]^	>100	>100	0
12	14**b** ^[b]^	>100	>100	0
13	14**c** ^[a]^	>100	>100	0
14	14**c** ^[b]^	>100	>100	0
15	14**d** ^[a]^	37	>100	>2.7
16	14**d** ^[b]^	54	74	1.4
17	Ribavirin^[a]^	3.2	>100	>31
18	Ribavirin^[b]^	3.1	42	14

[a] Control assay: visual (cytopathic effect/toxicity). [b] Control assay: neutral red (cytopathic effect/toxicity). [c] Drug control range 0.1–100 *μ*g/mL; ribavirin control concentration range 0.1–100 *μ*g/mL.
