# Supplementary material for: #Nitrosocarbonyls 1: Antiviral Activity of N-(4-Hydroxycyclohex-2-en-1-yl)quinoline-2-carboxamide against the Influenza A Virus H1N1
Source: ScientificWorldJournal. 2014 Dec 31;2014:472373. doi: 10.1155/2014/472373 (PMC4293787; doi:10.1155/2014/472373)
Supplement: Supplementary file 1 — 1. 1H and 13C NMR spectra. 2. Cartesian coordinates of calculated structures. [file 472373.f1.doc]

Supporting Information

**#Nitrosocarbonyls 1**

**Antiviral Activity of *N*-(4-hydroxycyclohex-2-en-1-yl)-quinoline-2-carboxamide against the Influenza A virus H1N1**

Dalya Al-Saad, Misal Giuseppe Memeo and Paolo Quadrelli*

Content

1. ^1^H and ^13^C NMR spectra Pag. S2
2. Cartesian coordinates of calculated structures Pag. S17

1. ^1^H and ^13^C NMR spectra


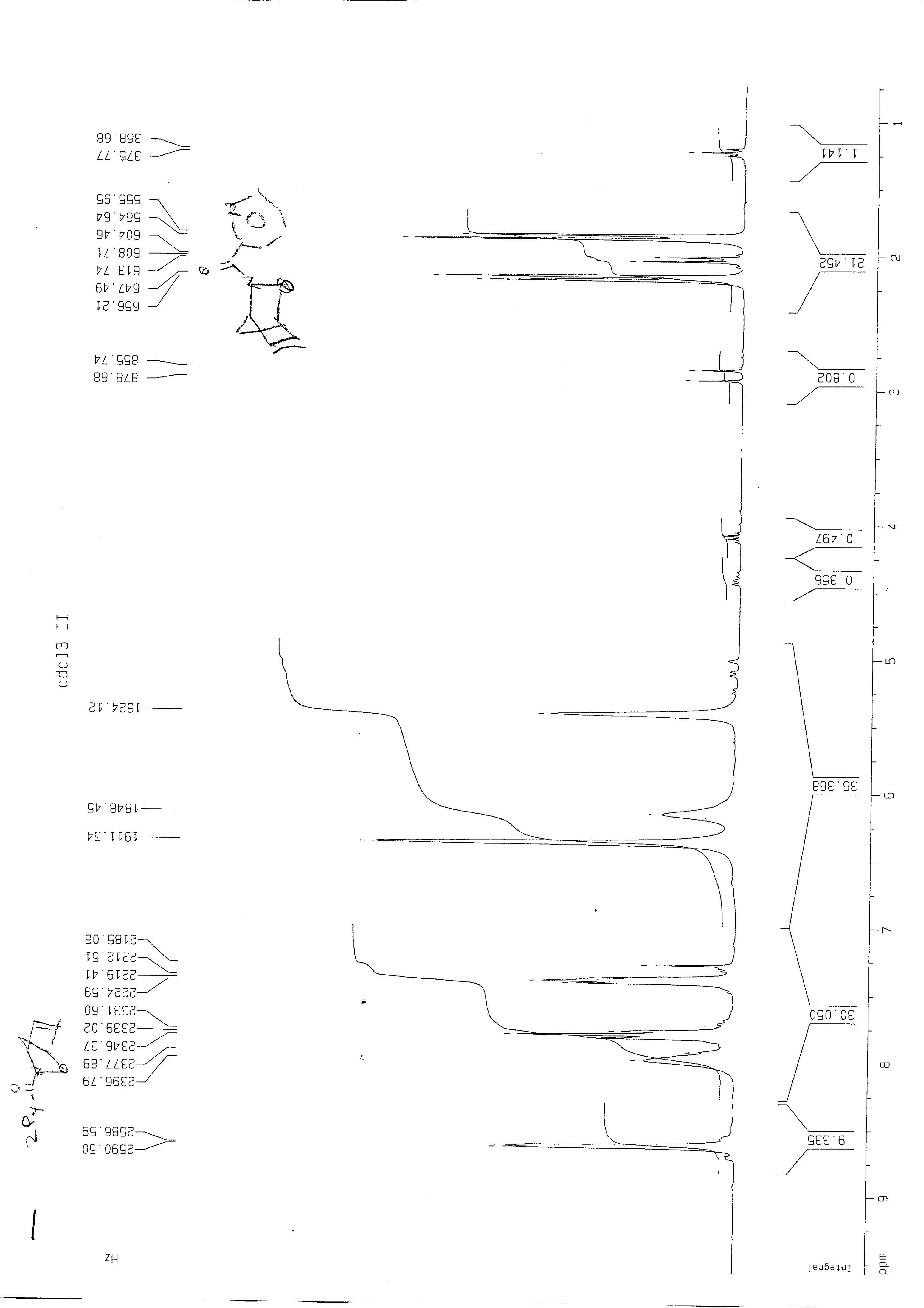
11a


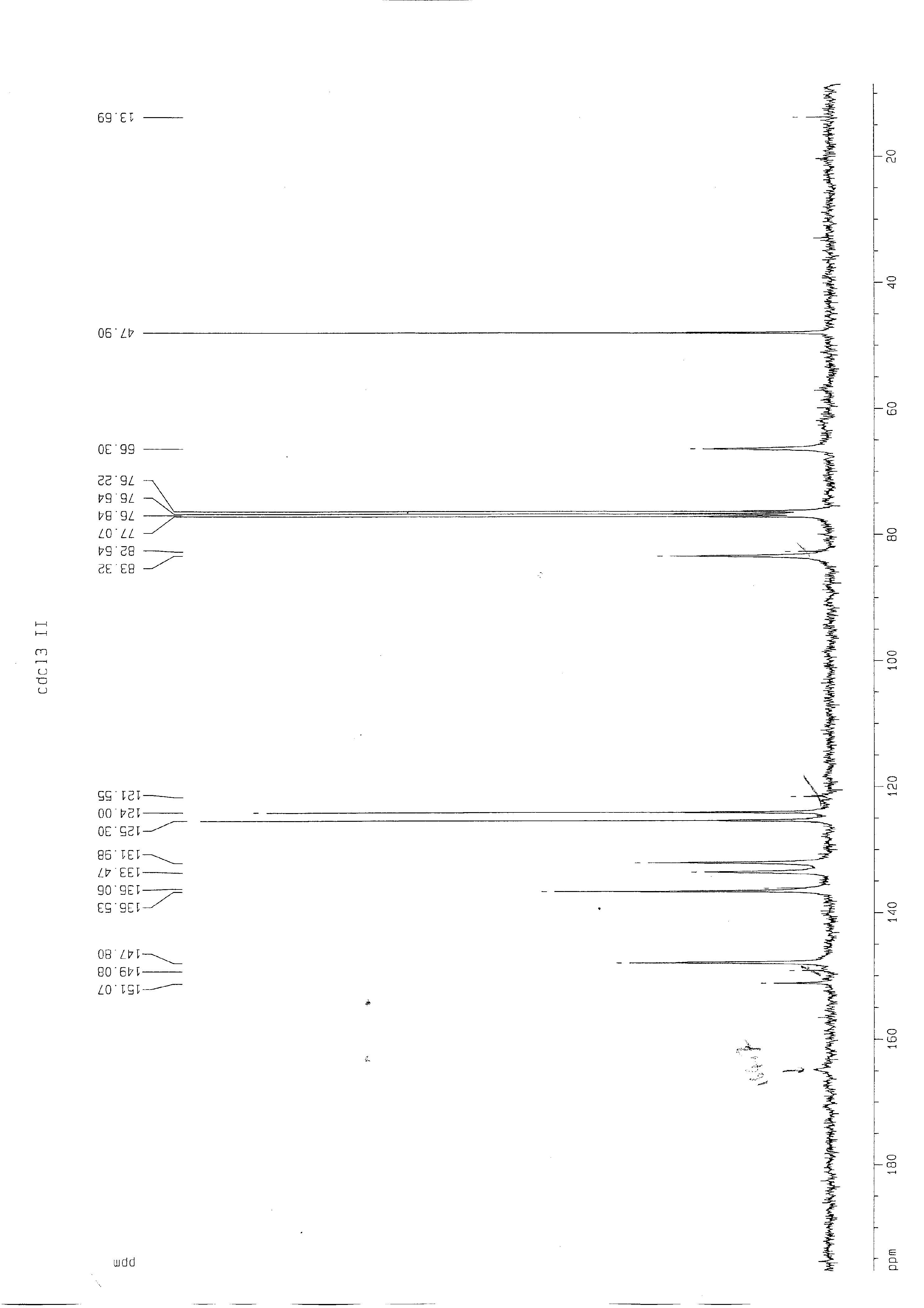


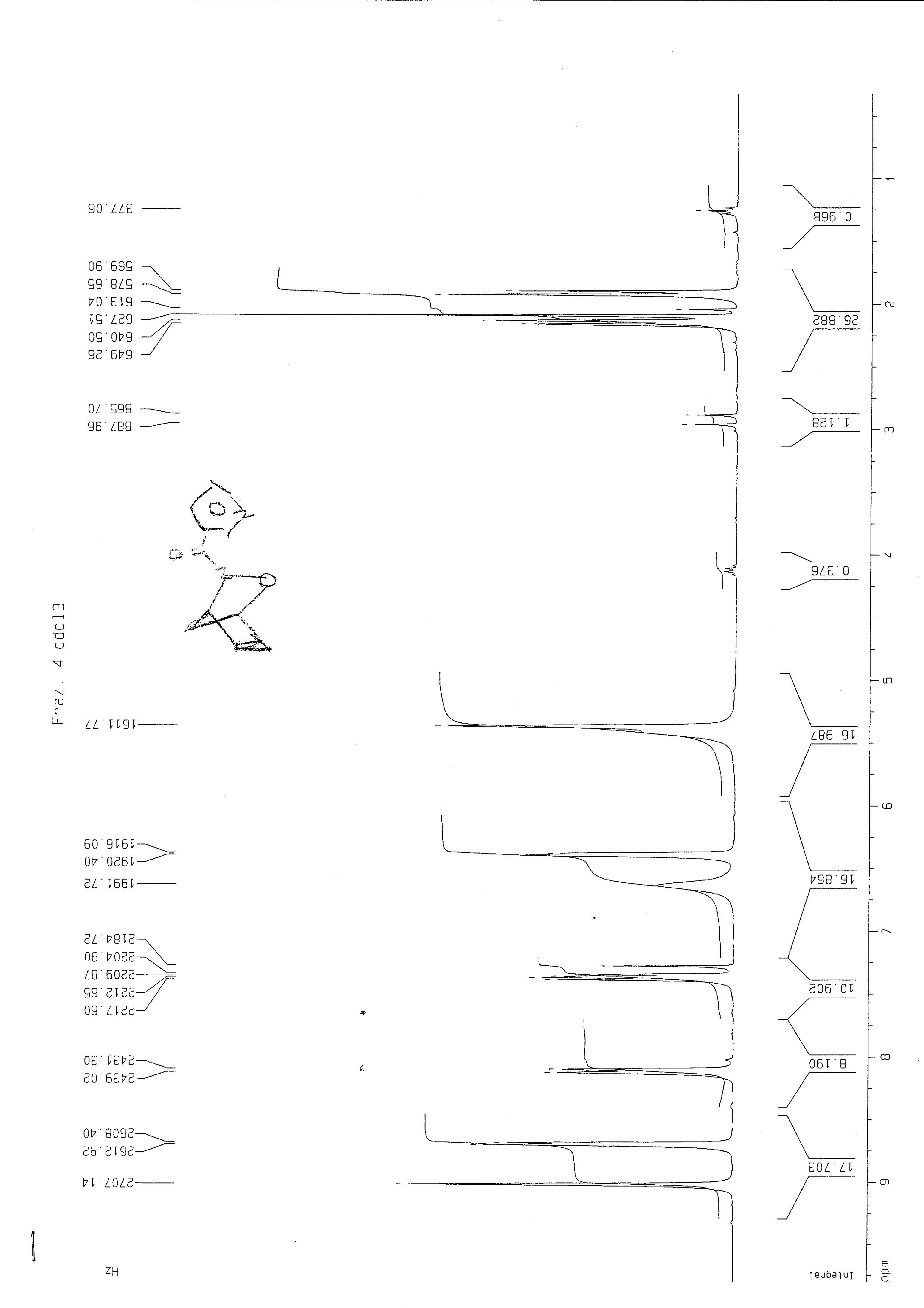

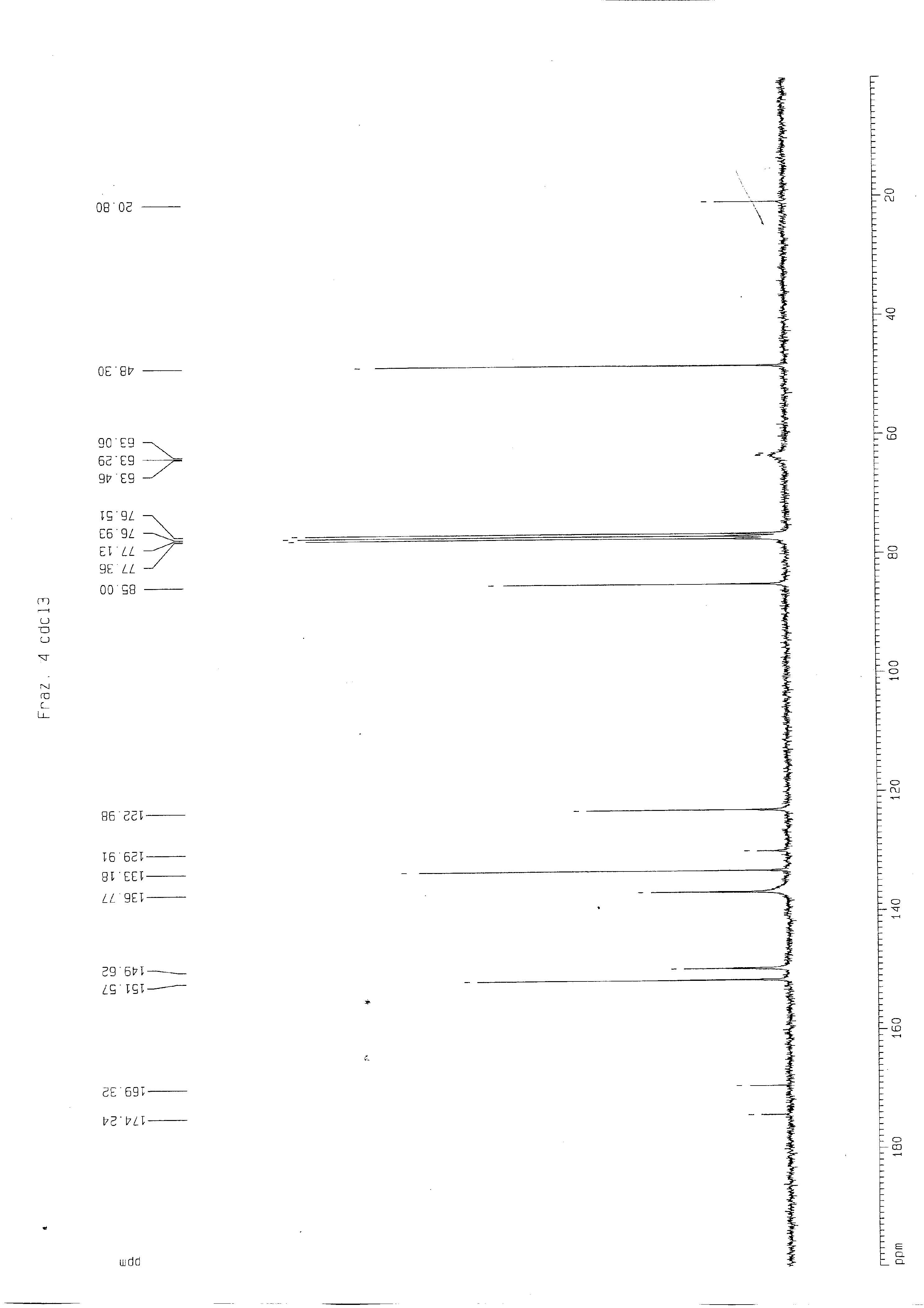


11b

11
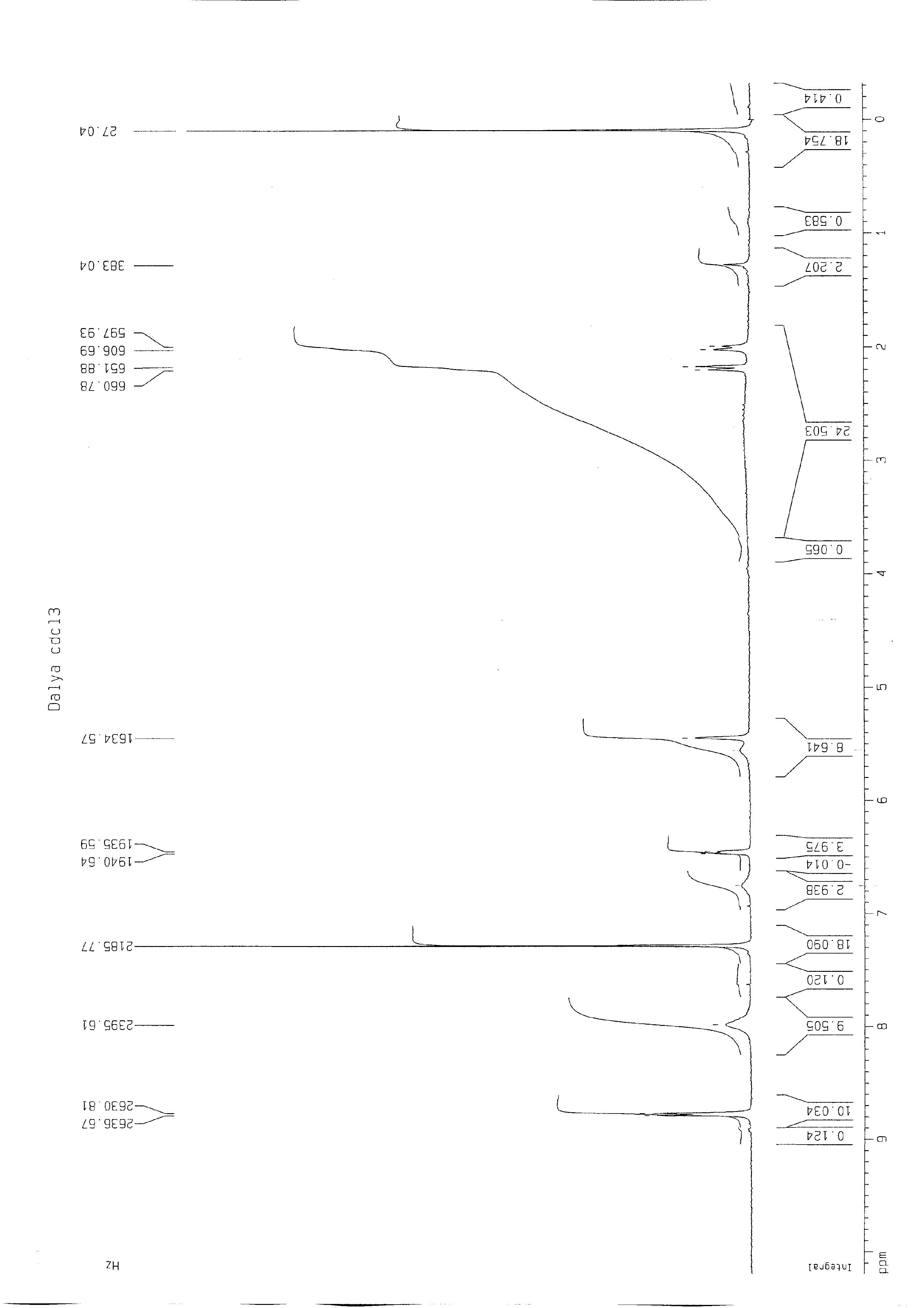

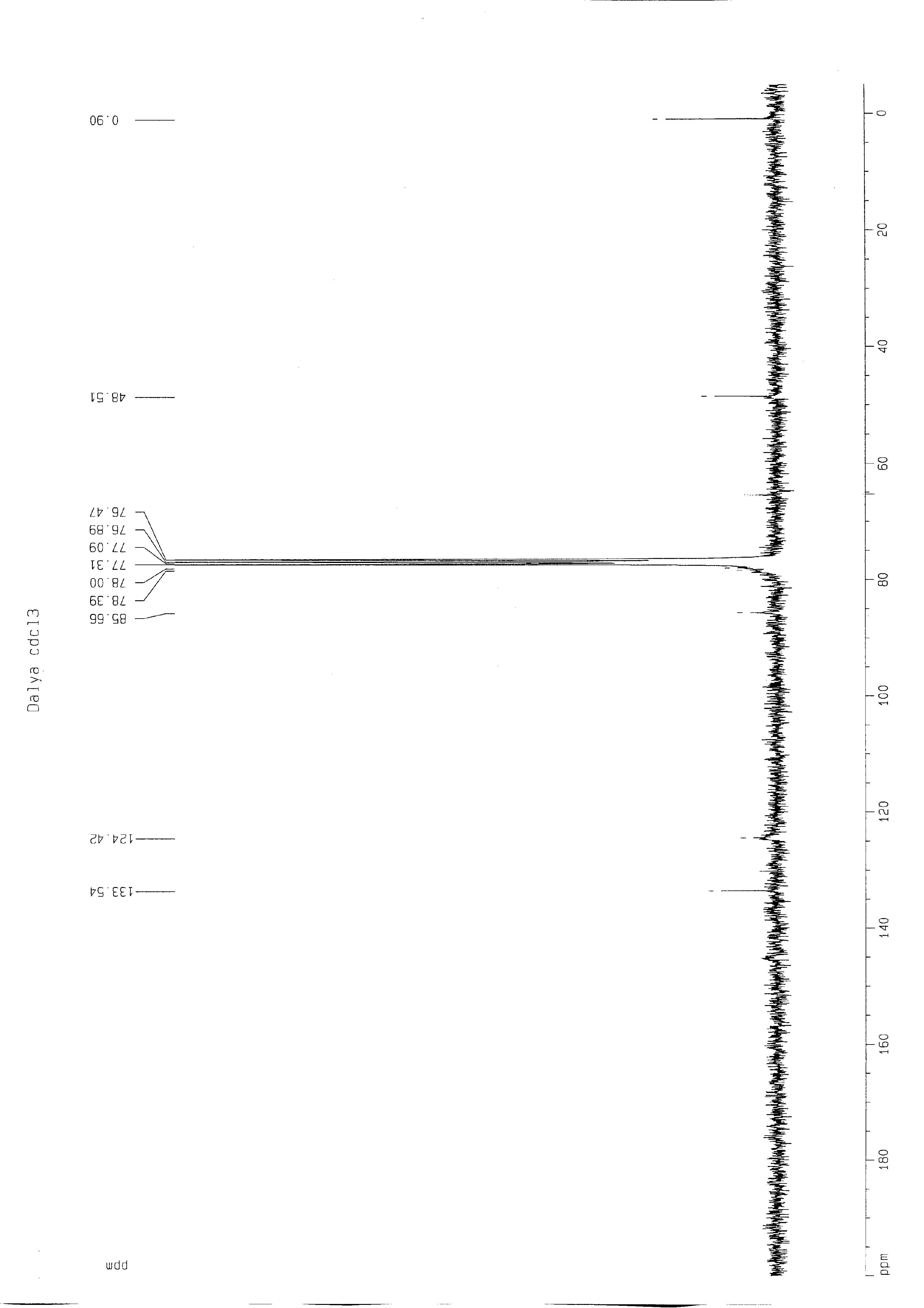
c


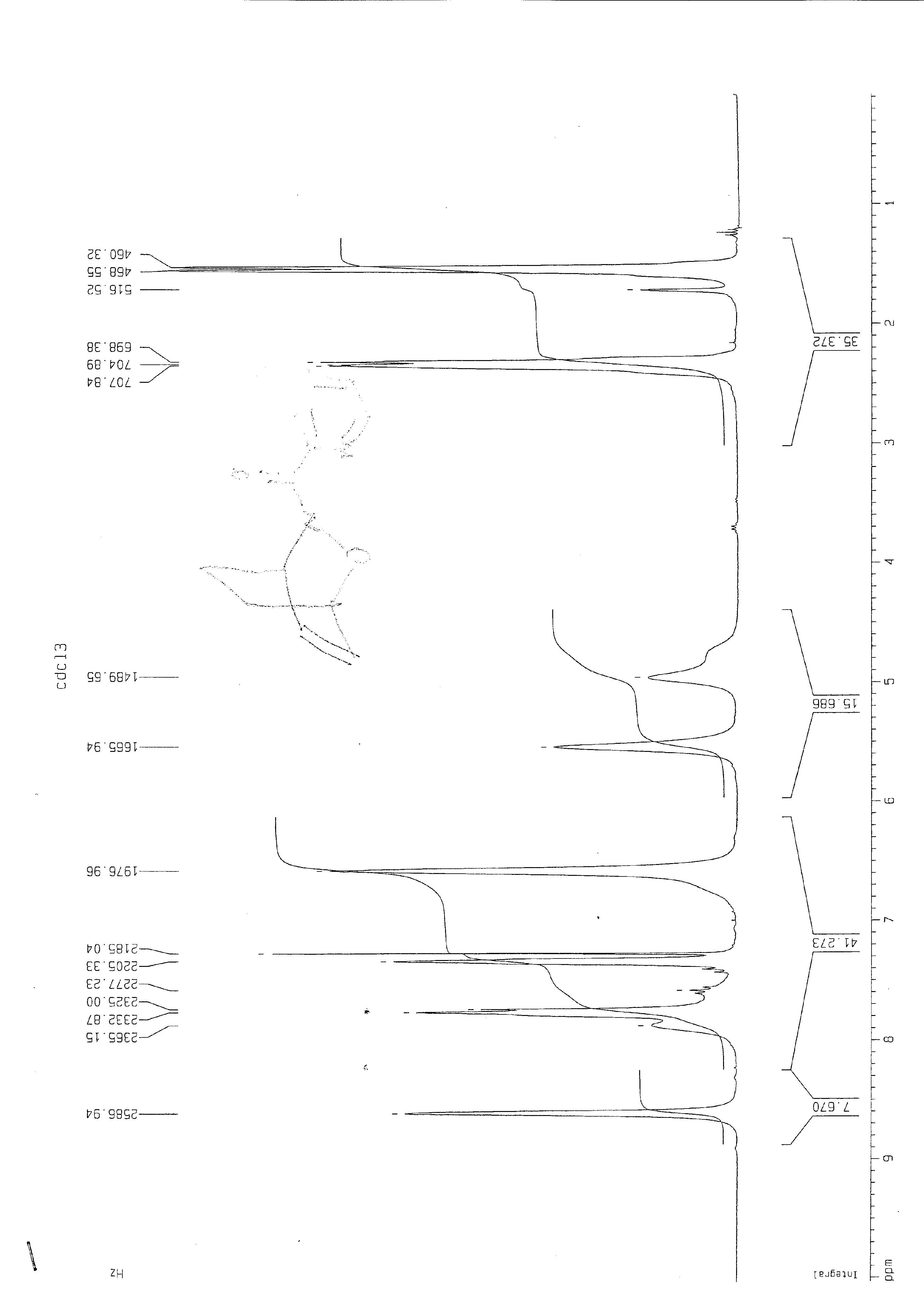


12a


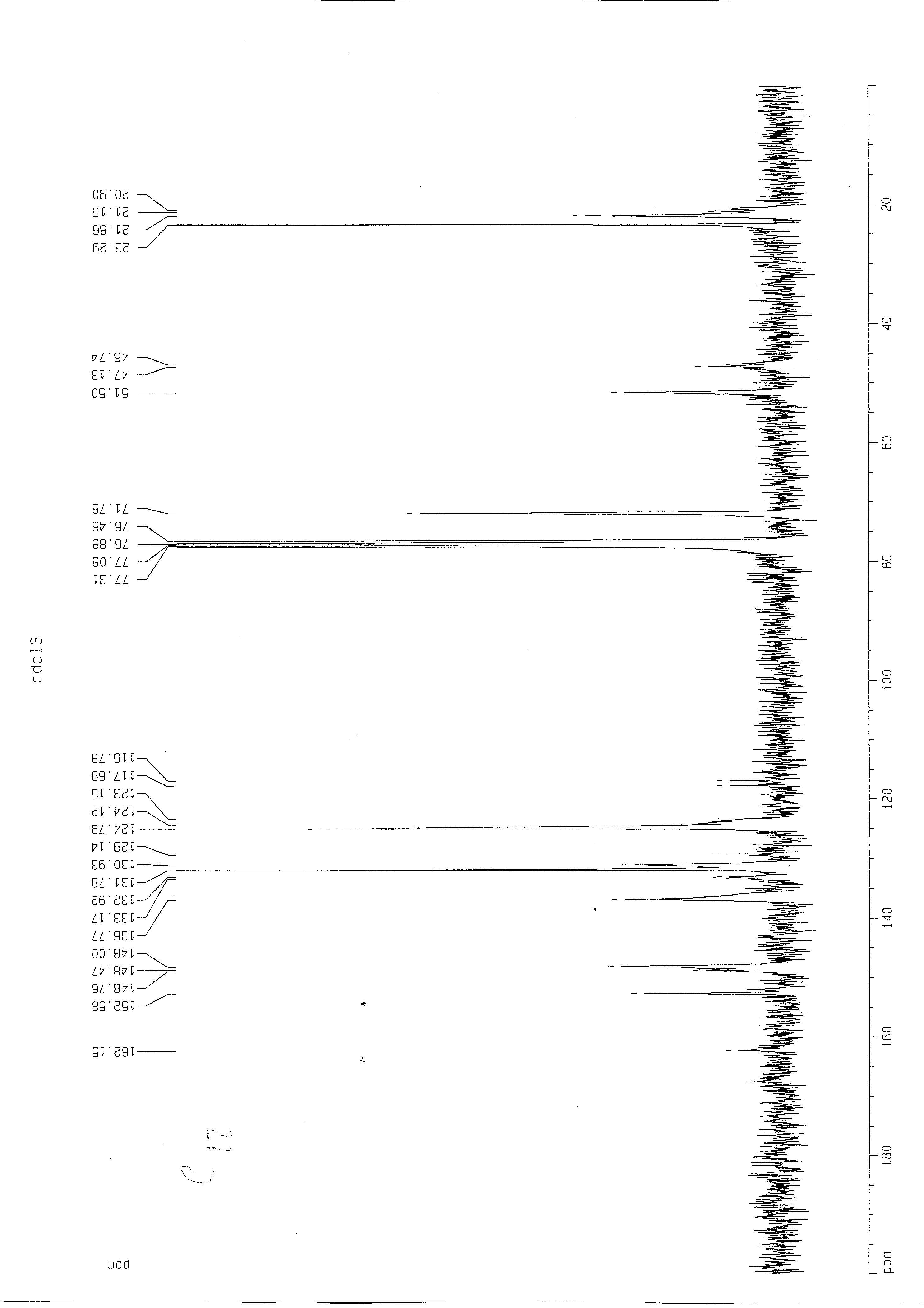


12
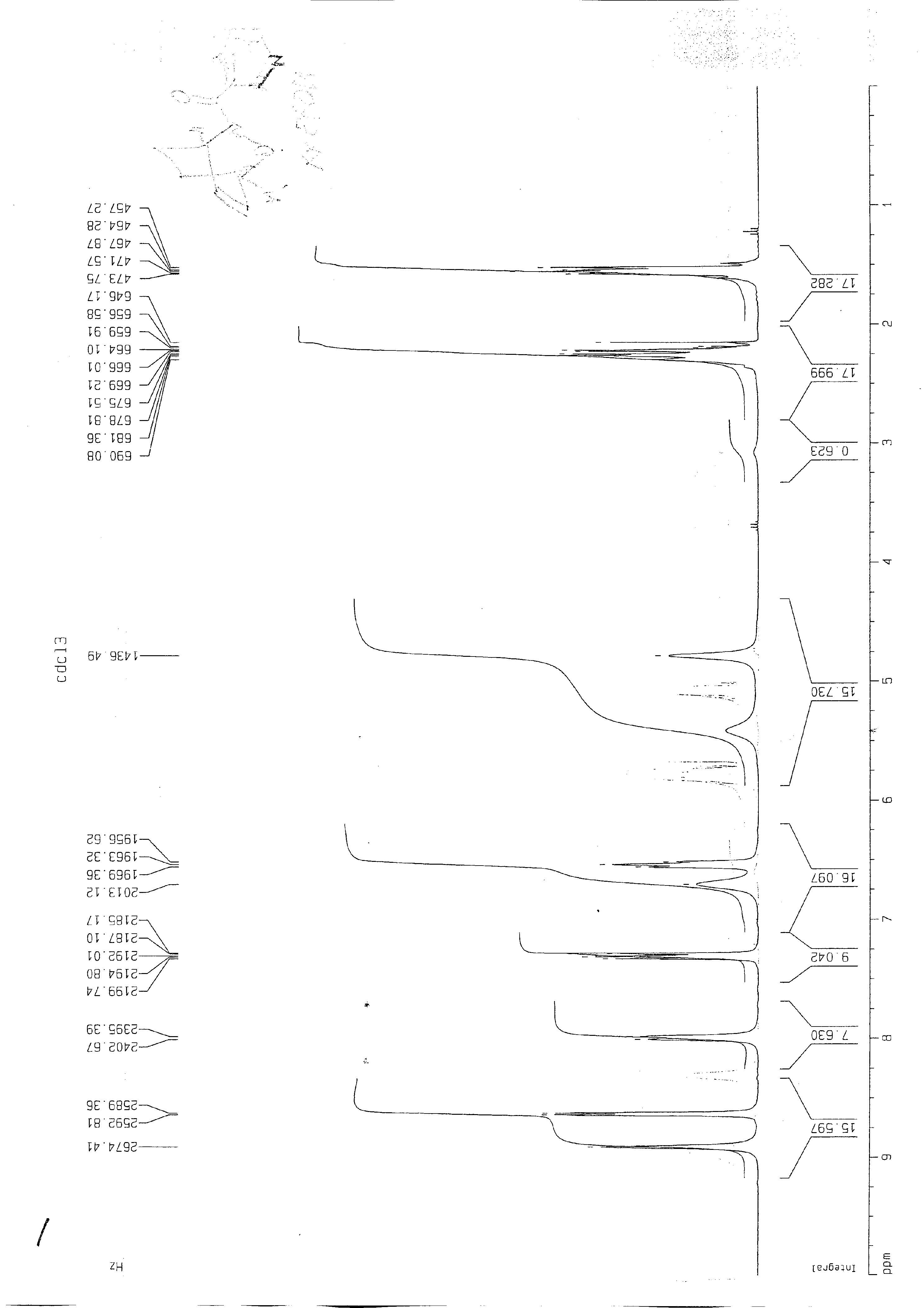

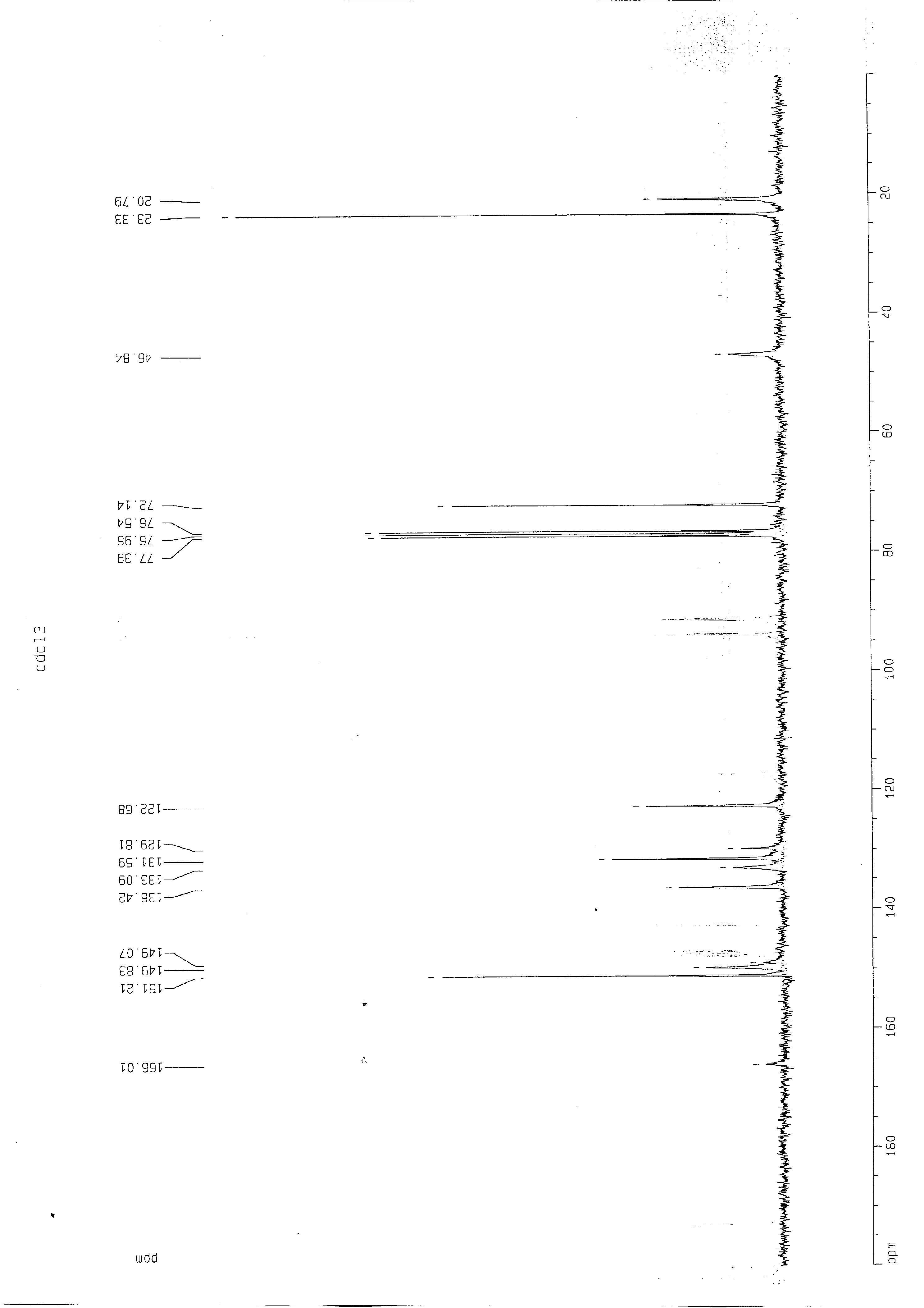
b

12
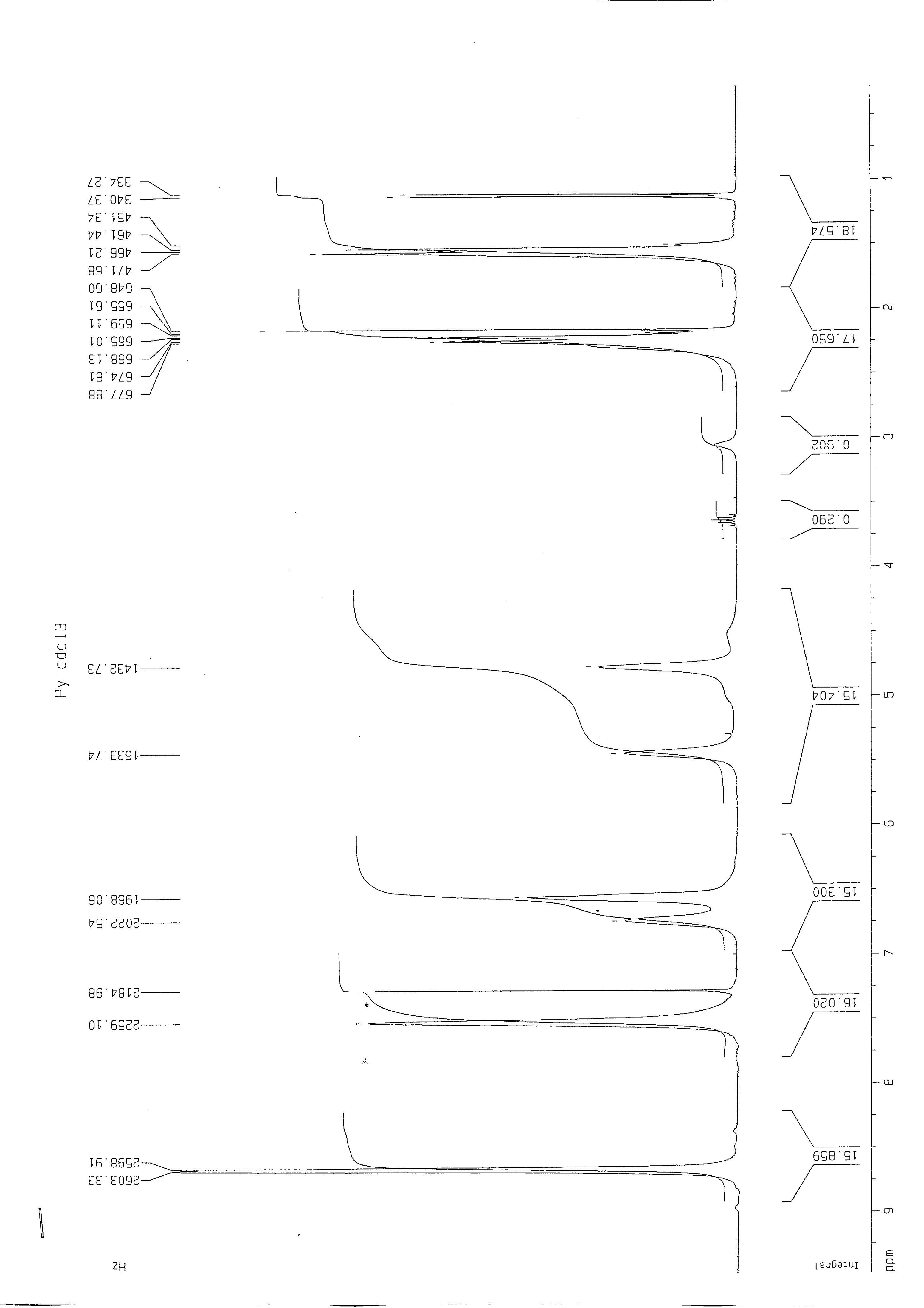
c


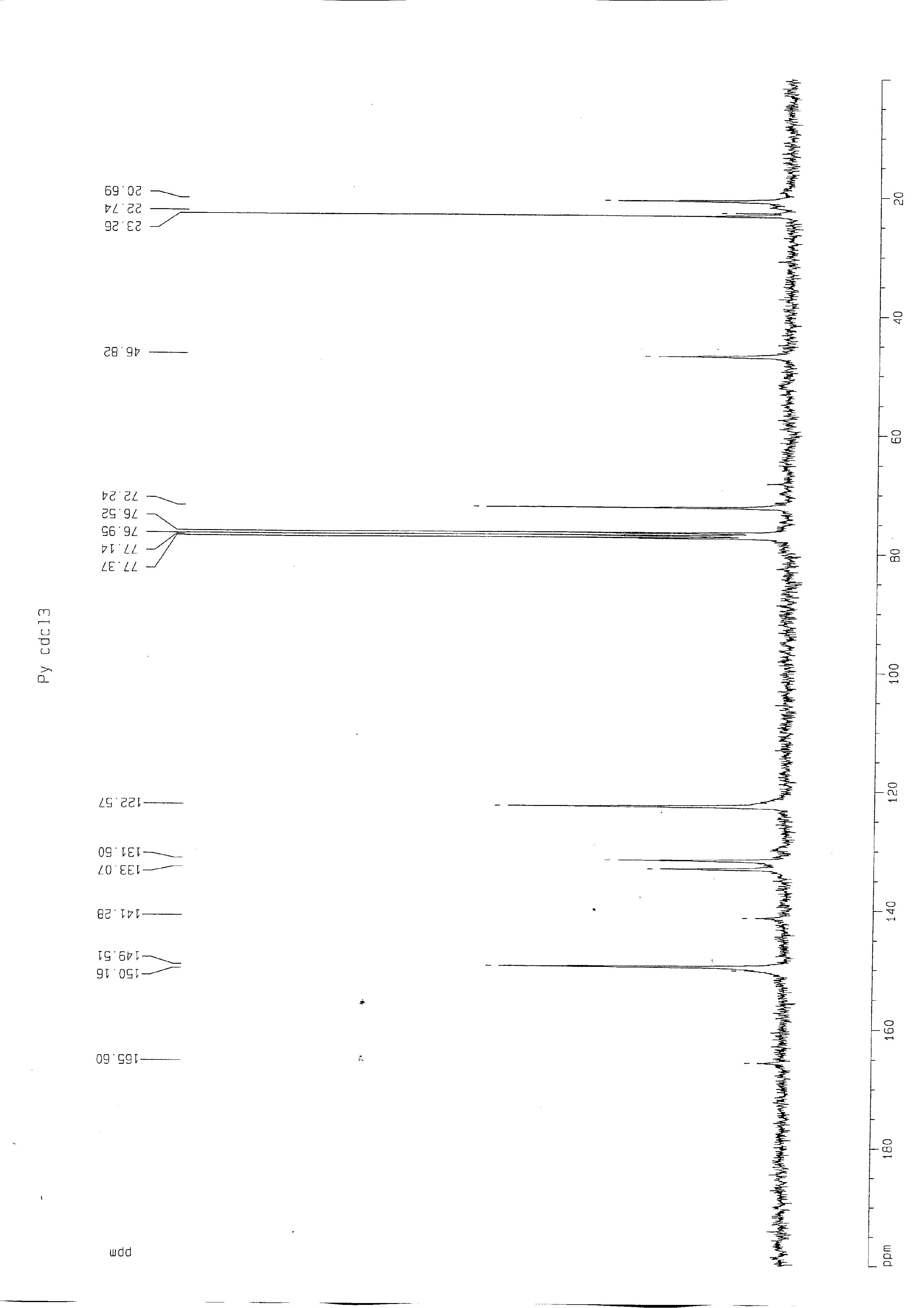


12
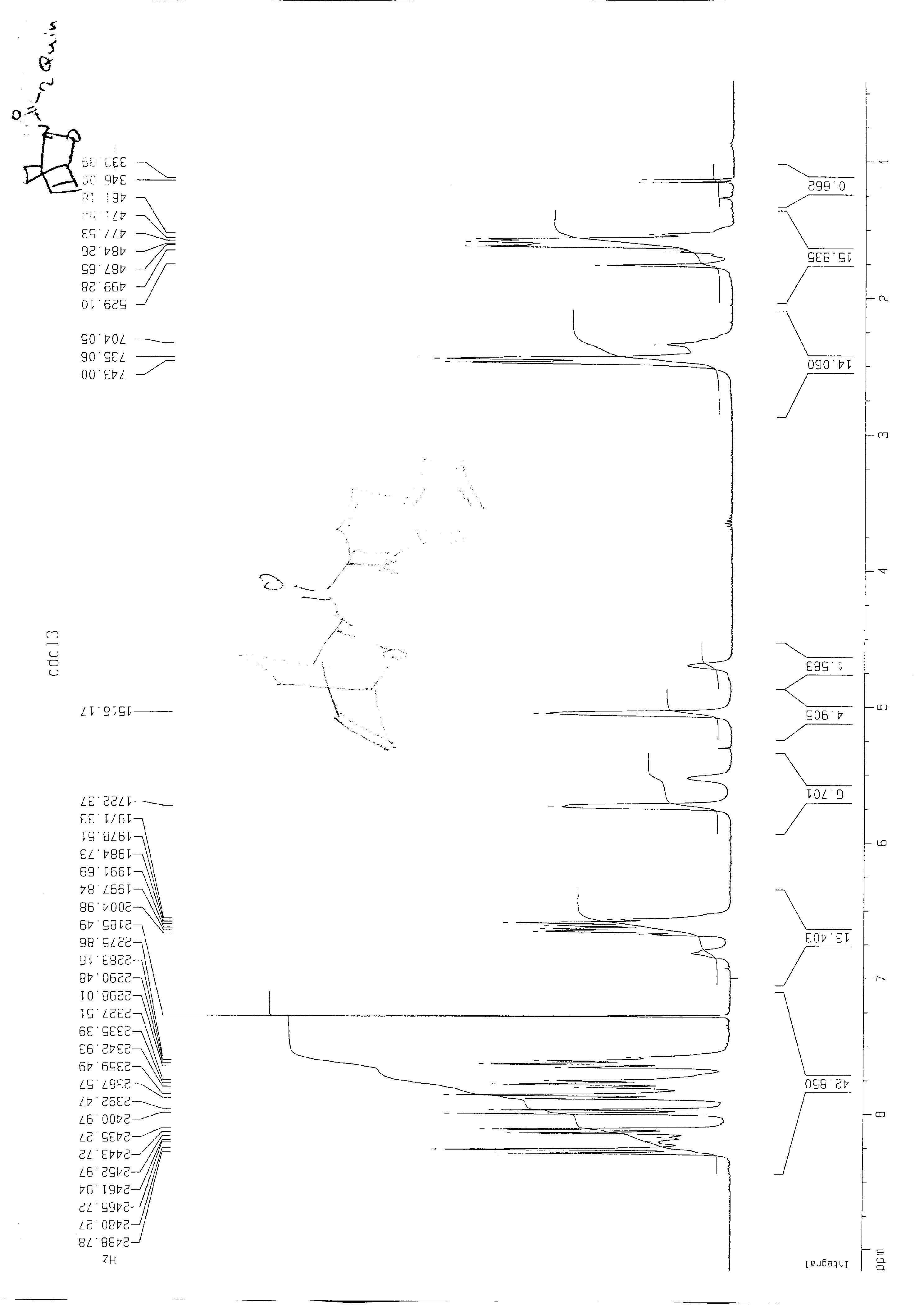

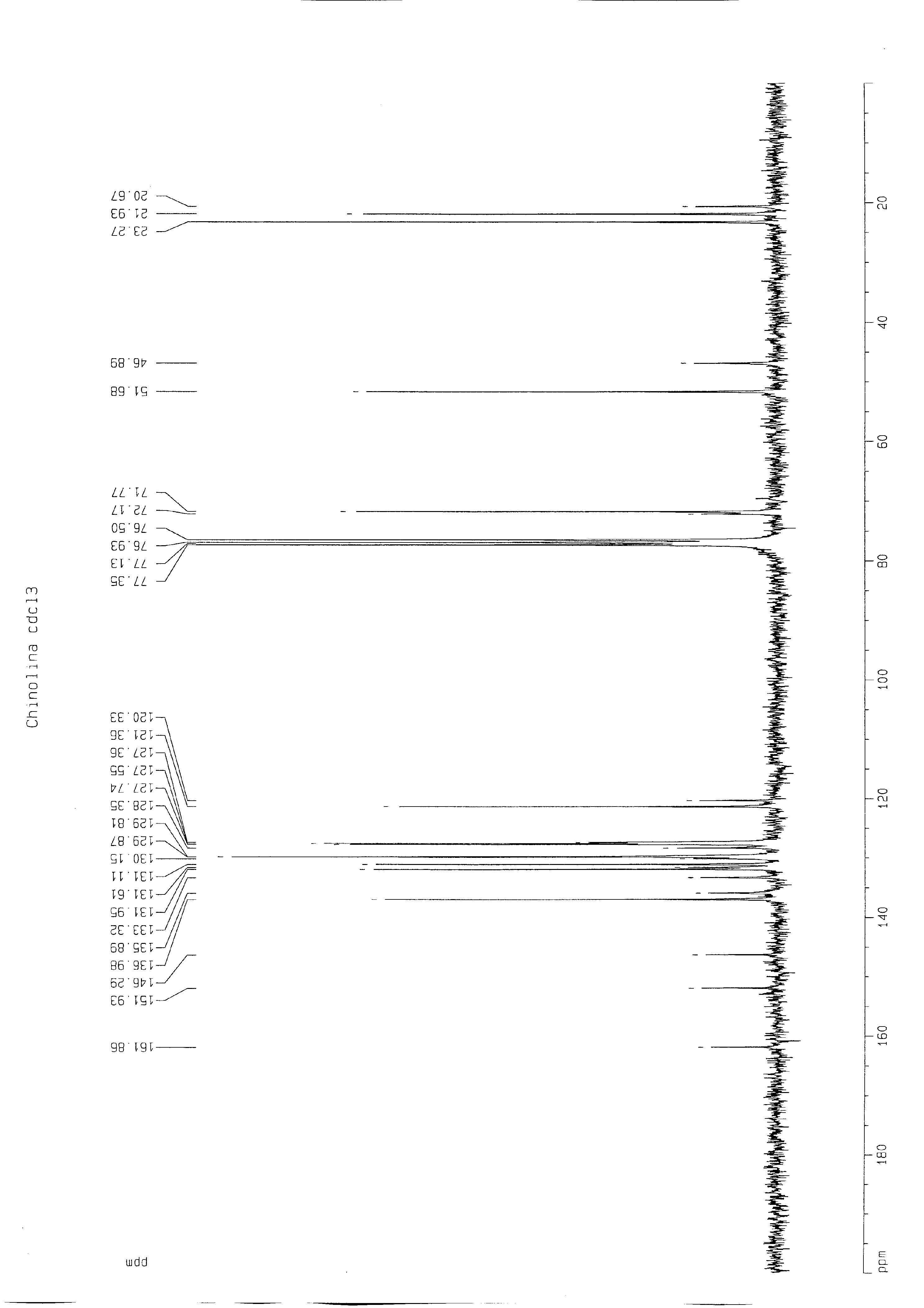
d


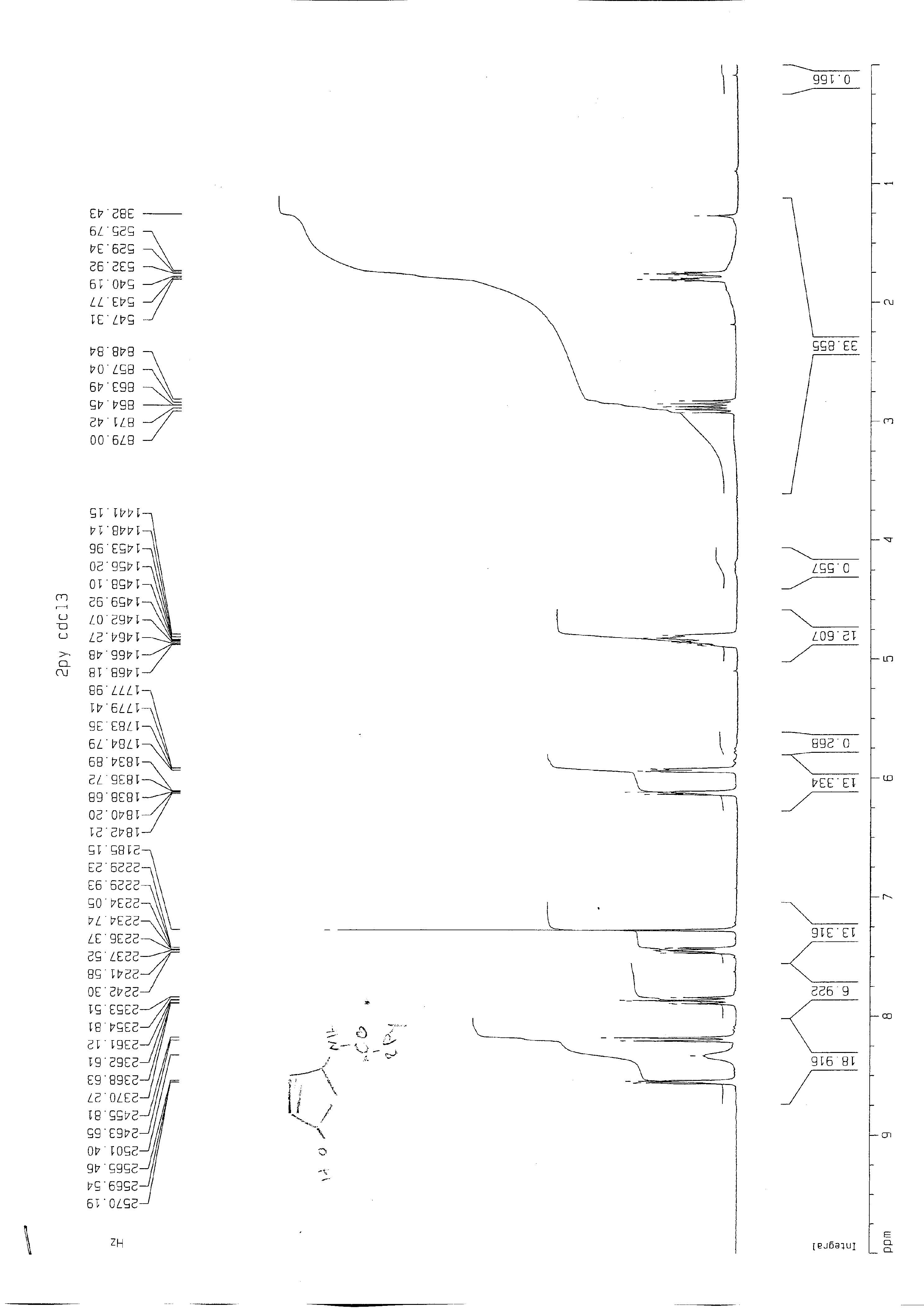

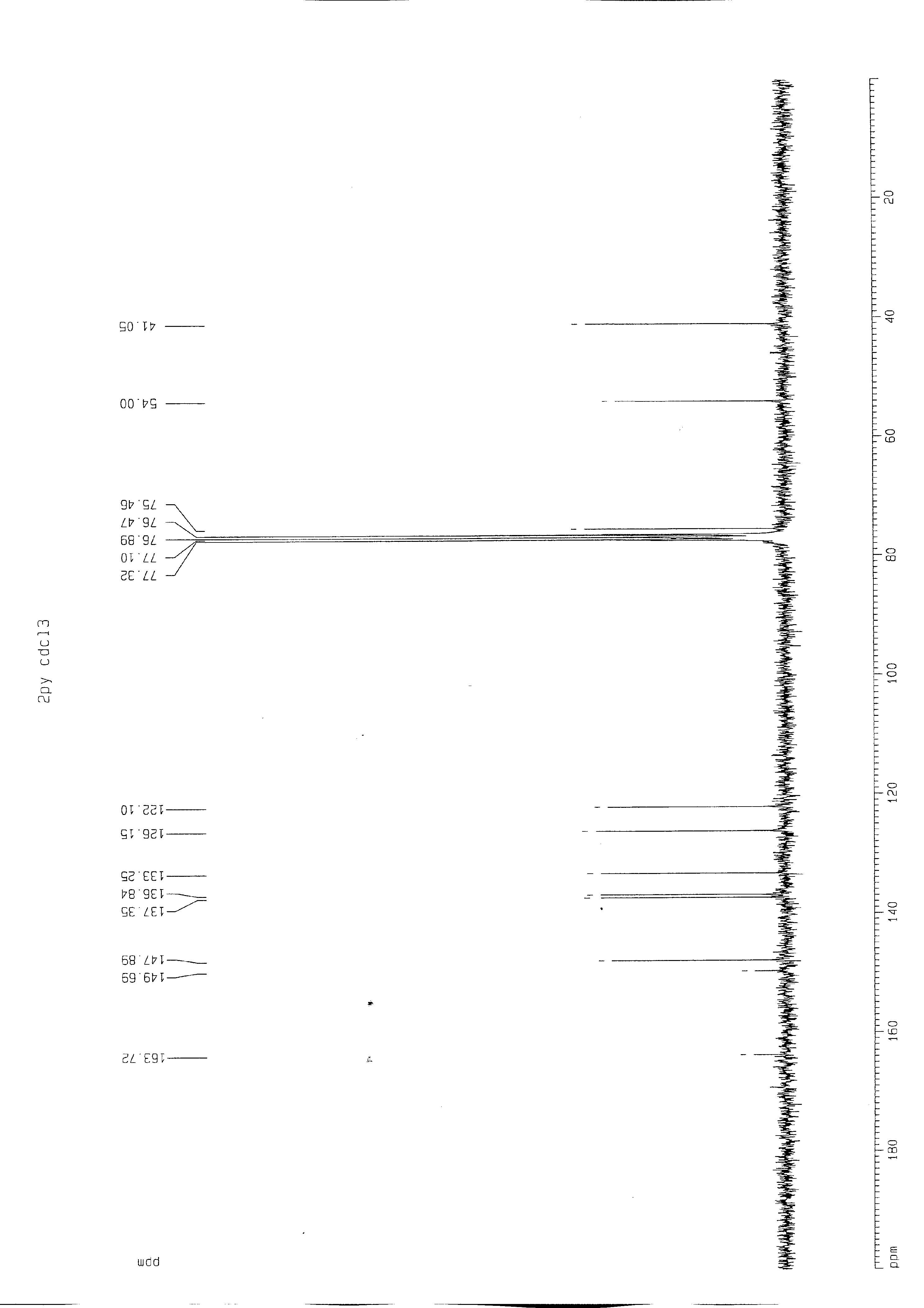
13a

1
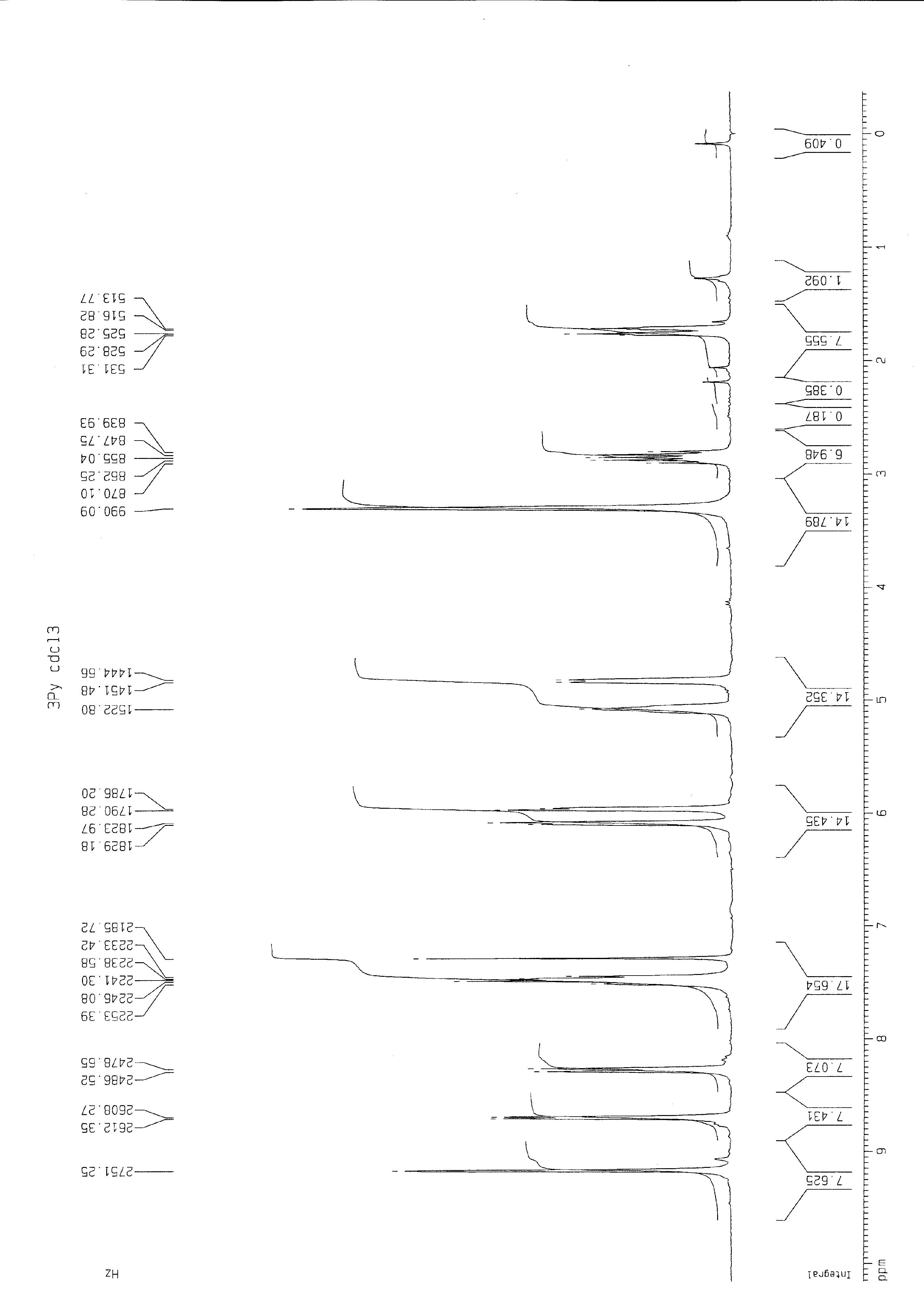

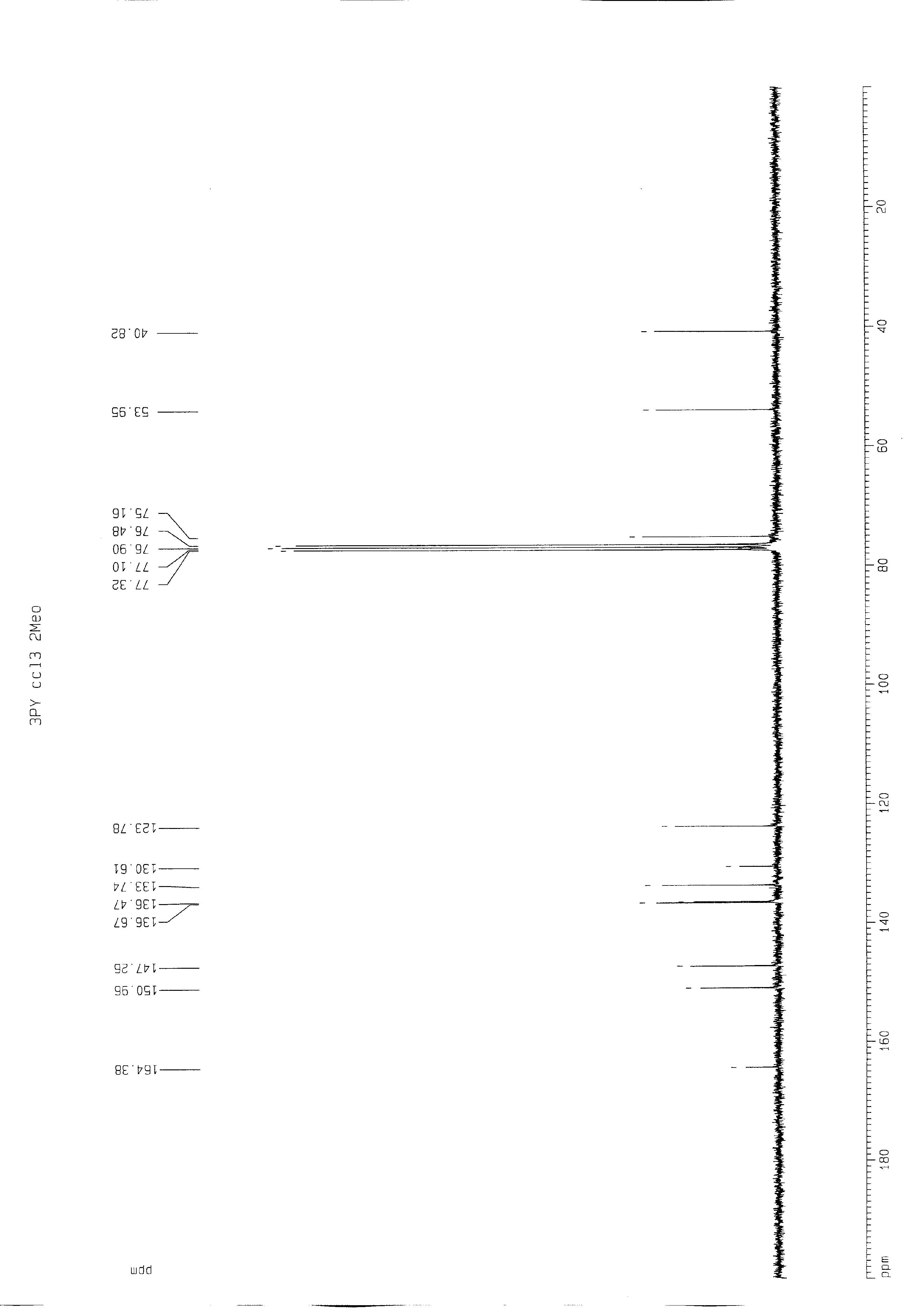
3b


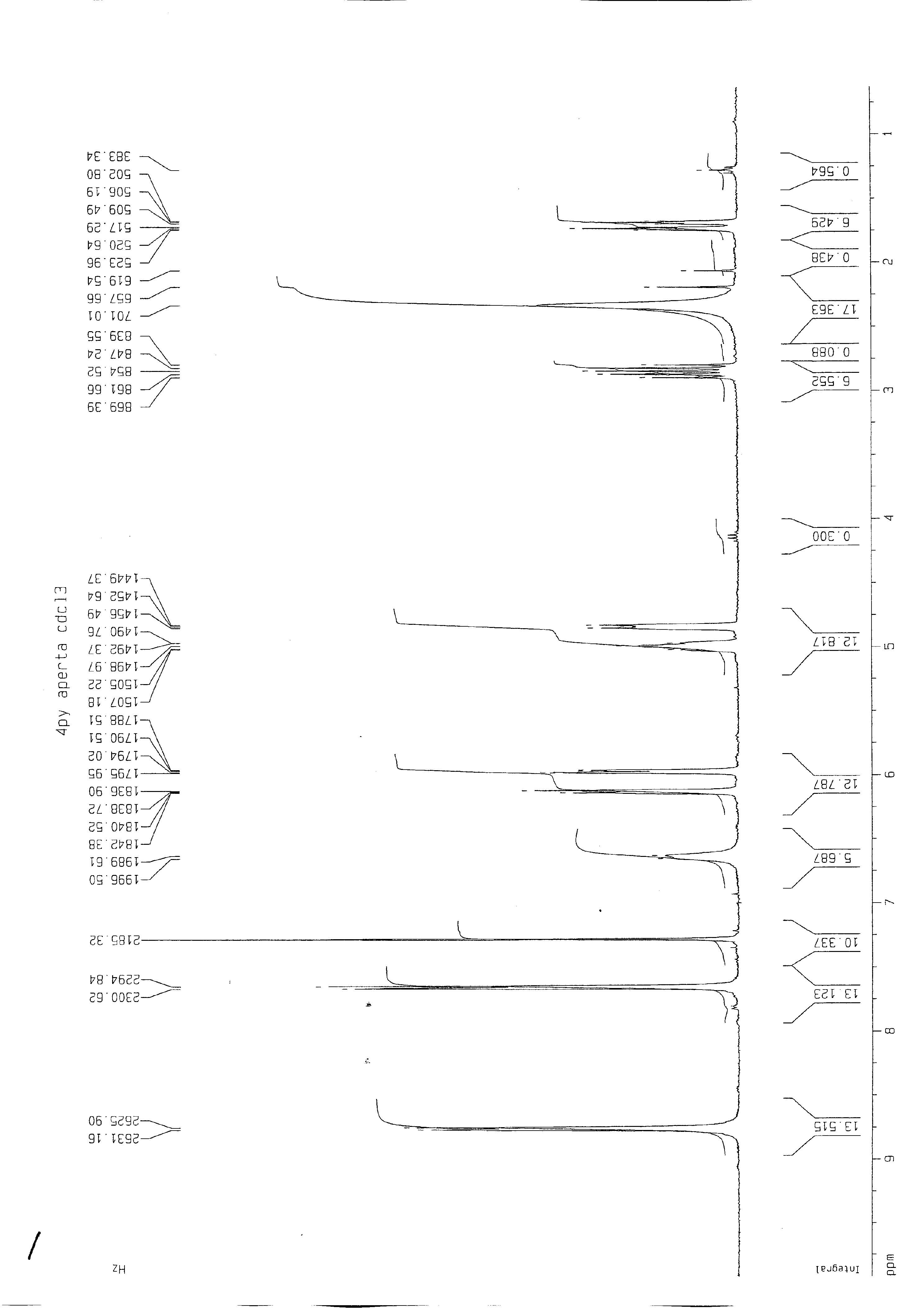


1
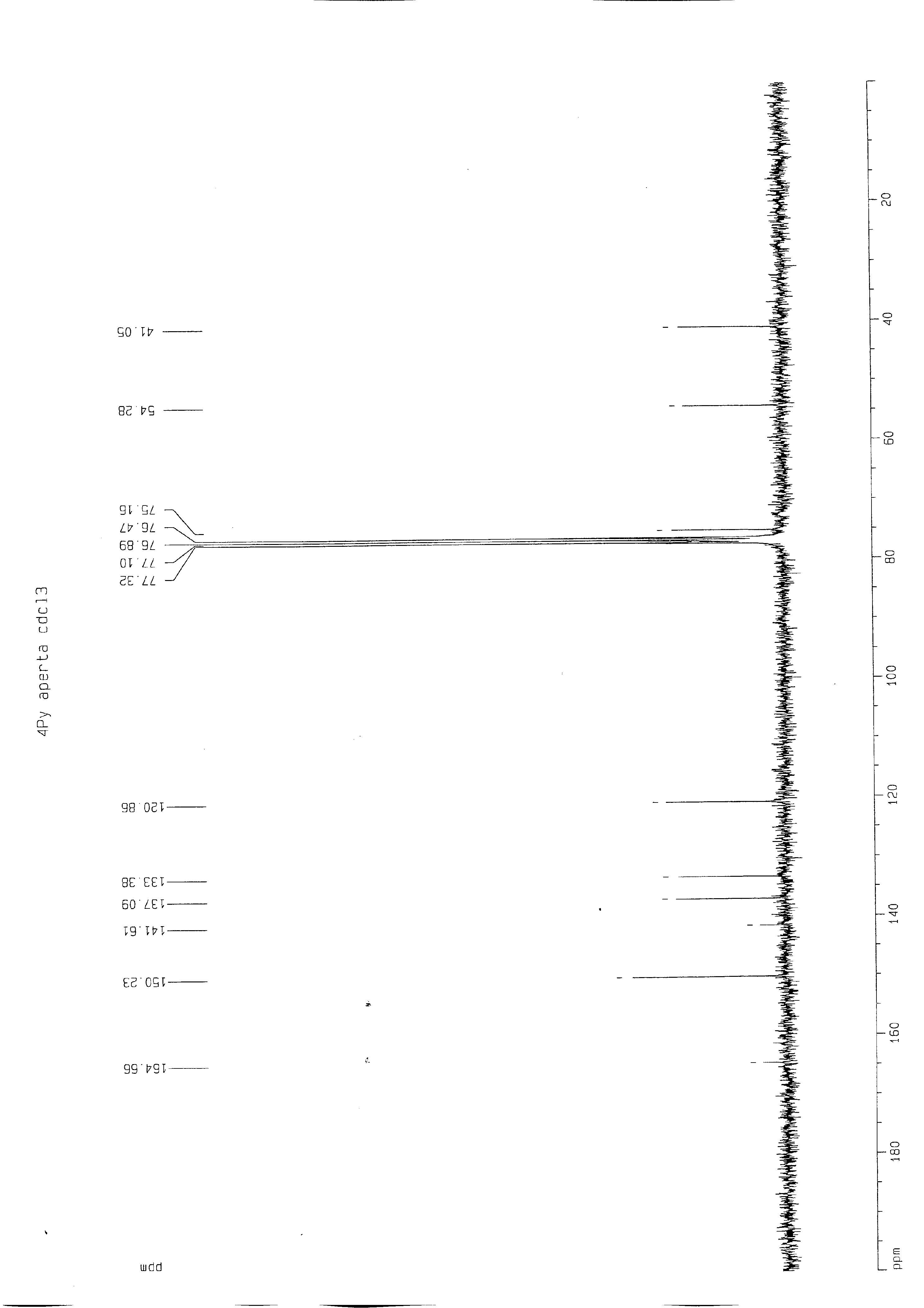
3c


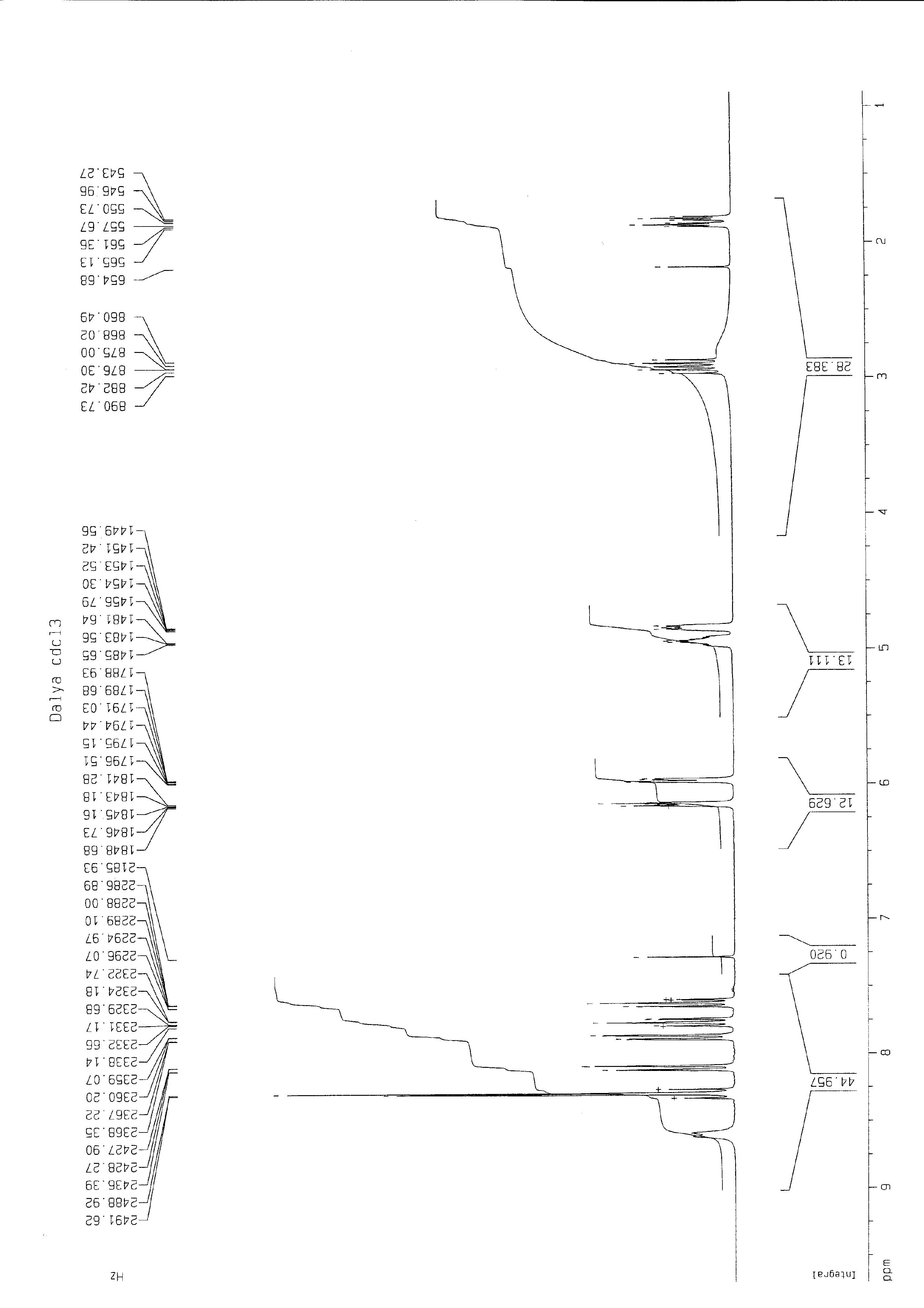

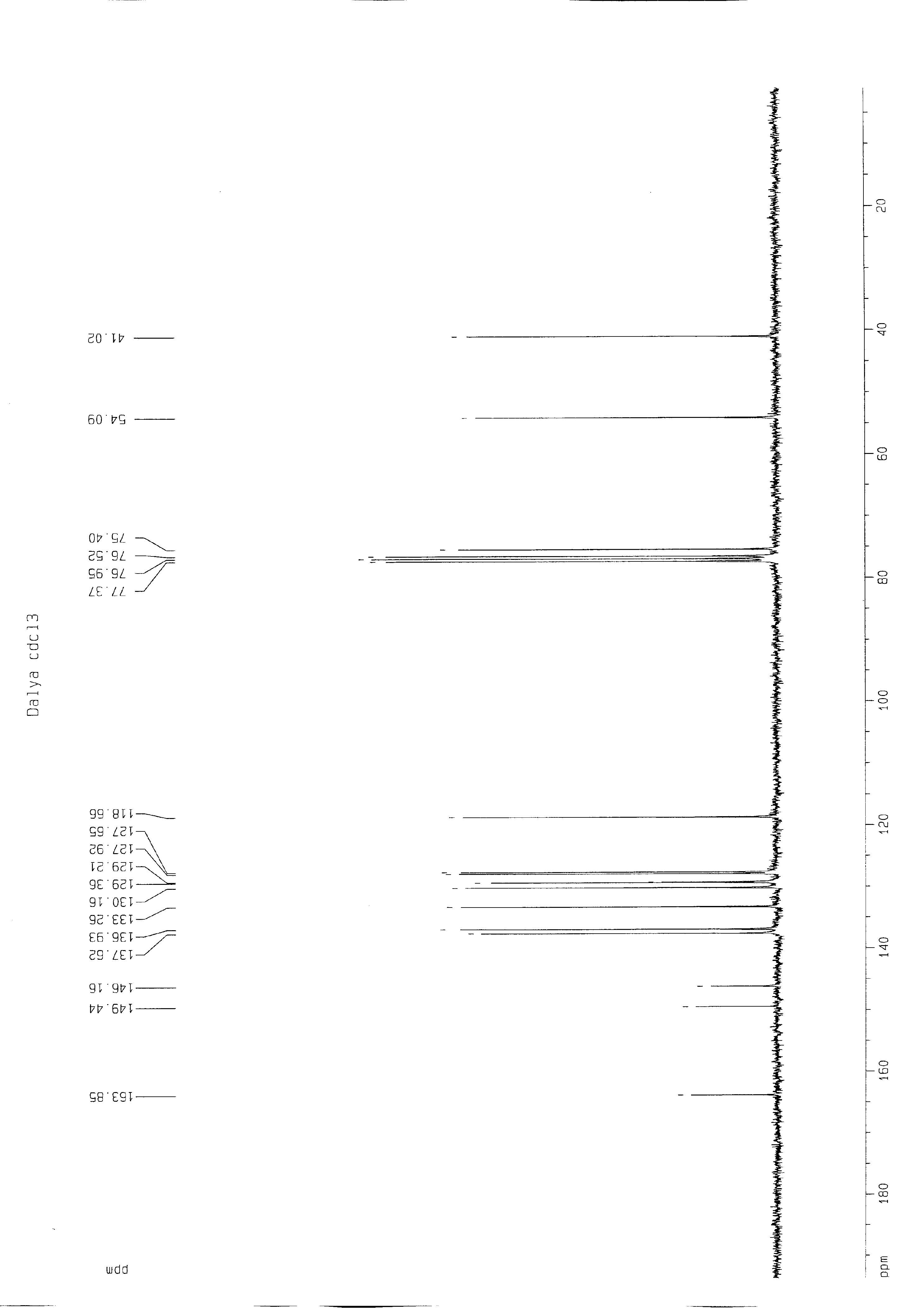


13d


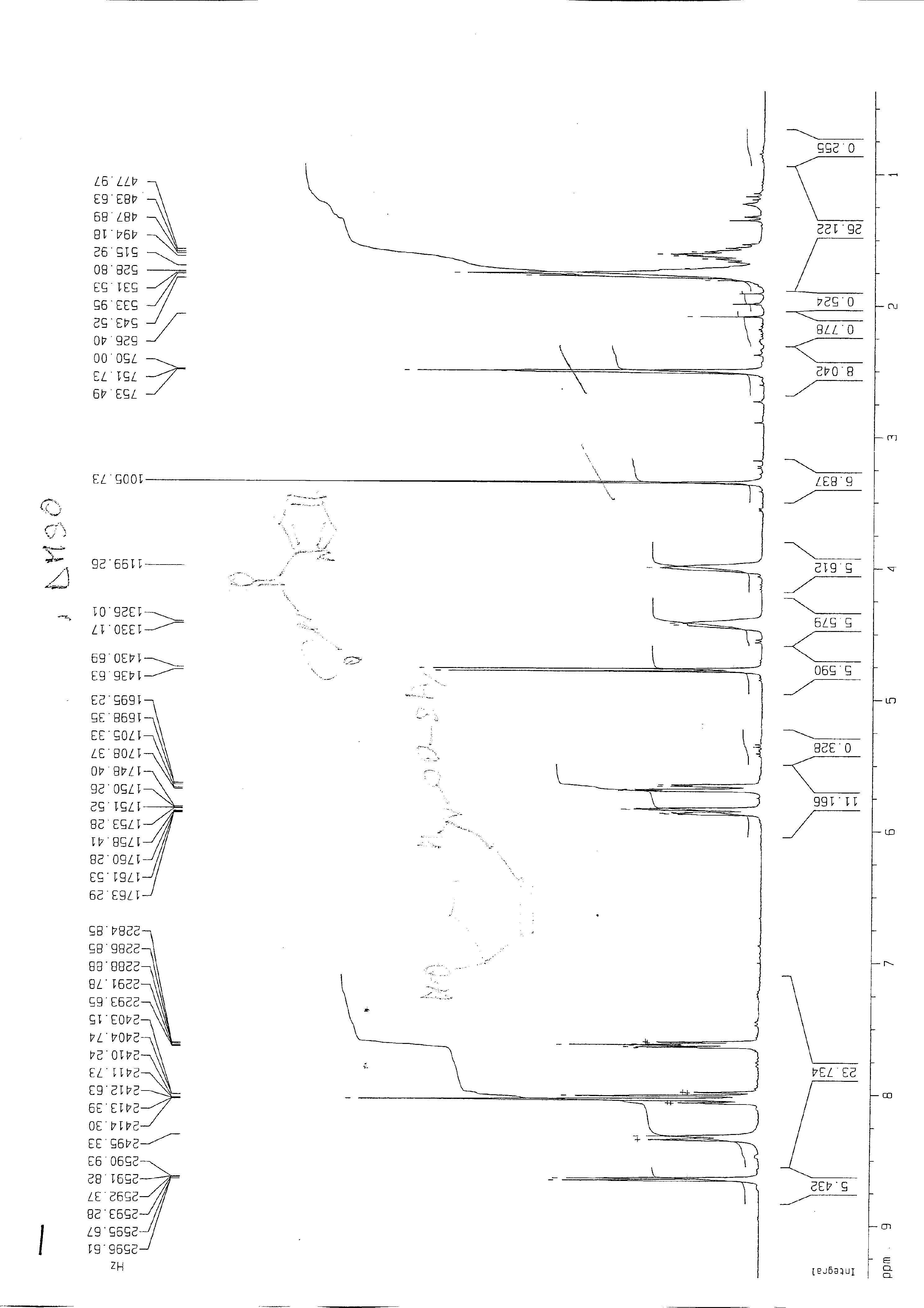


14a


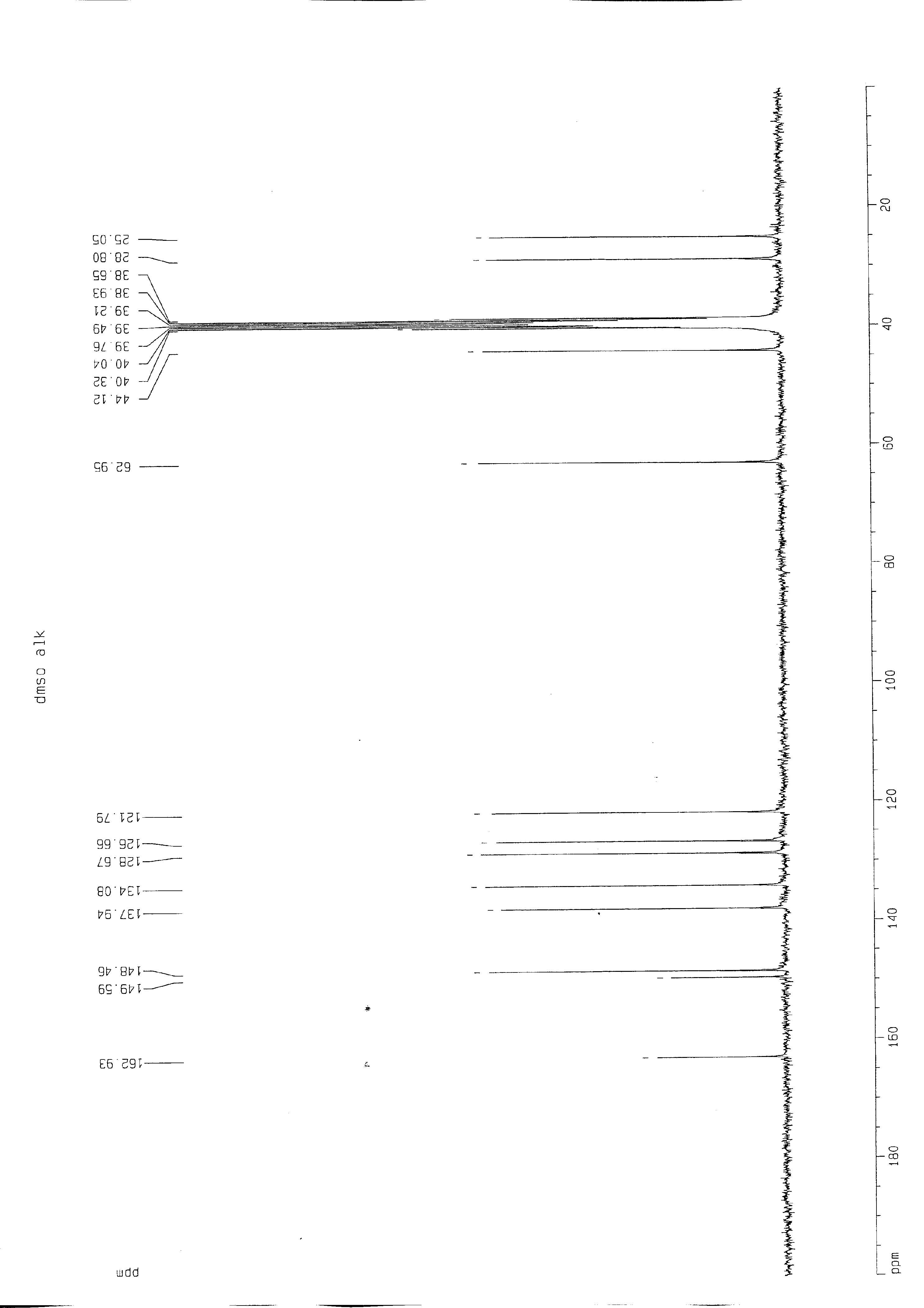


1
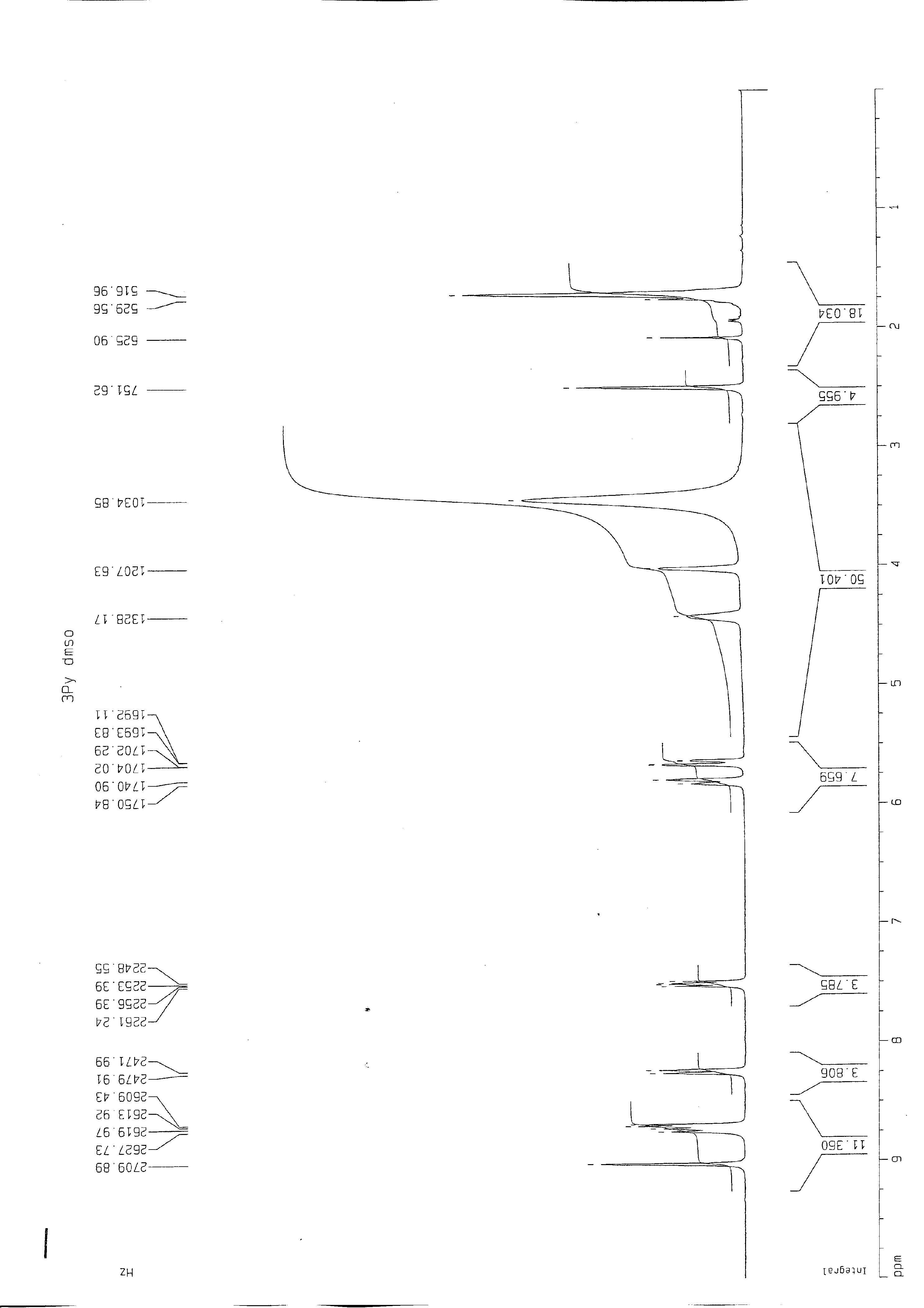
4b


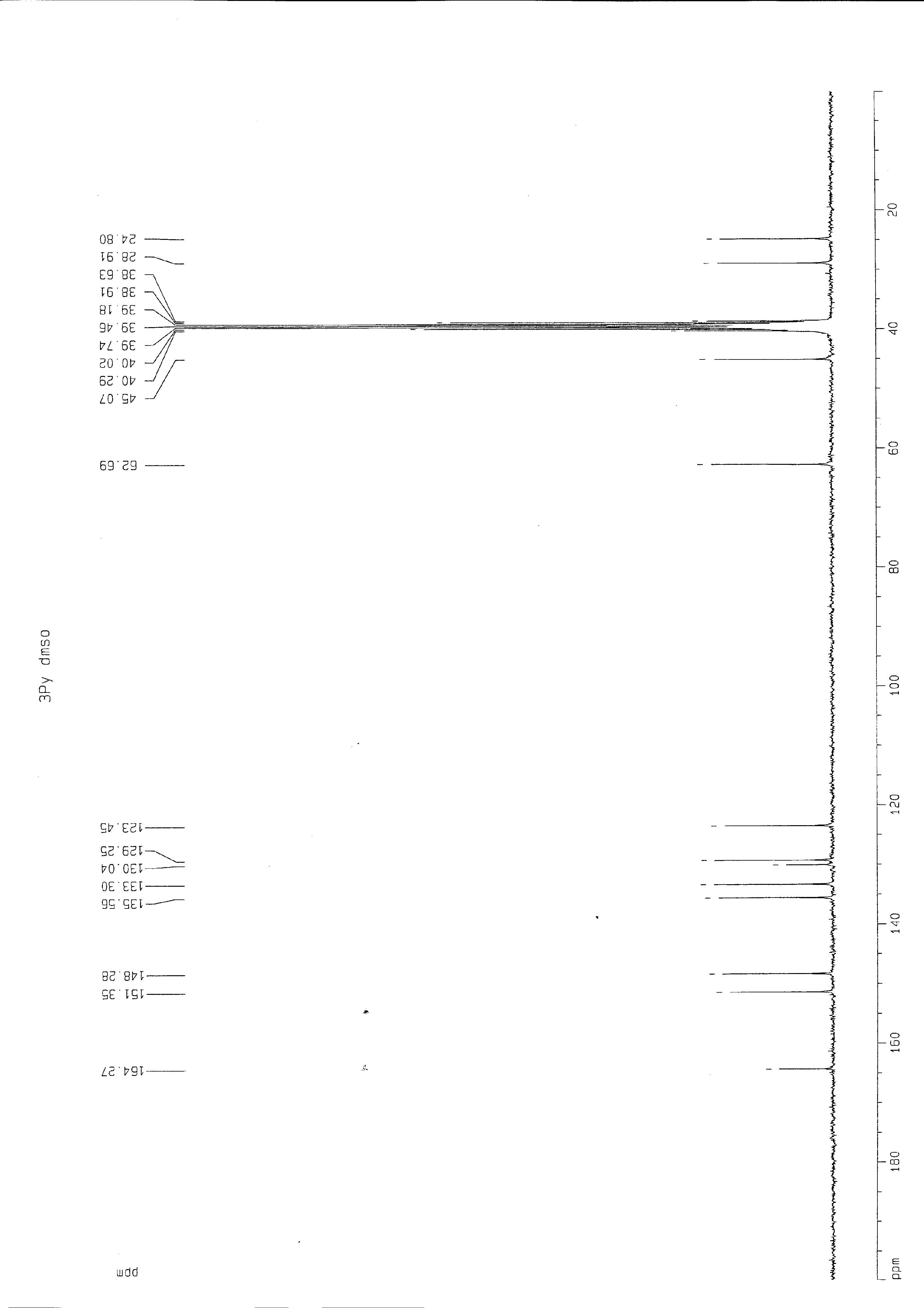


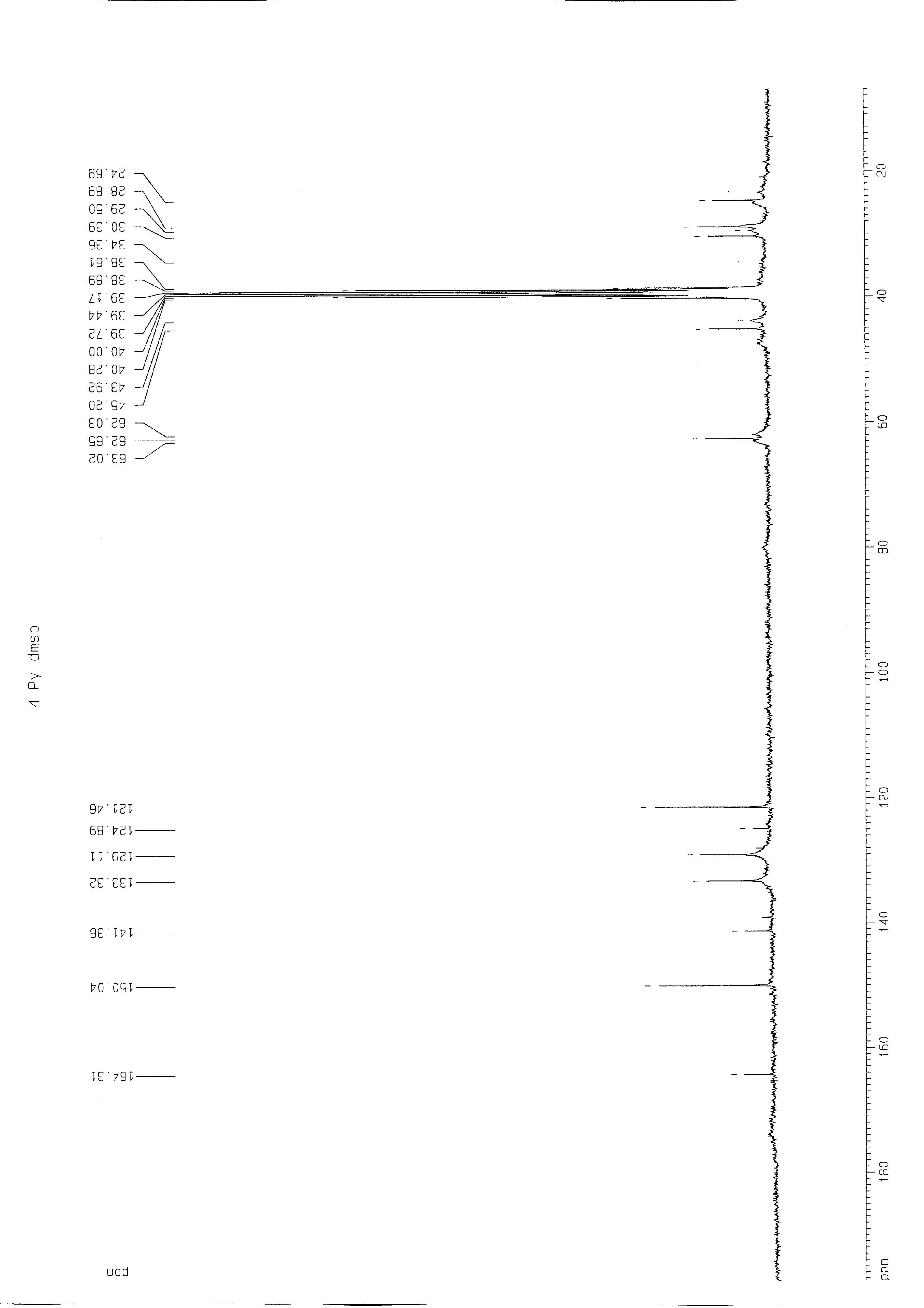
1
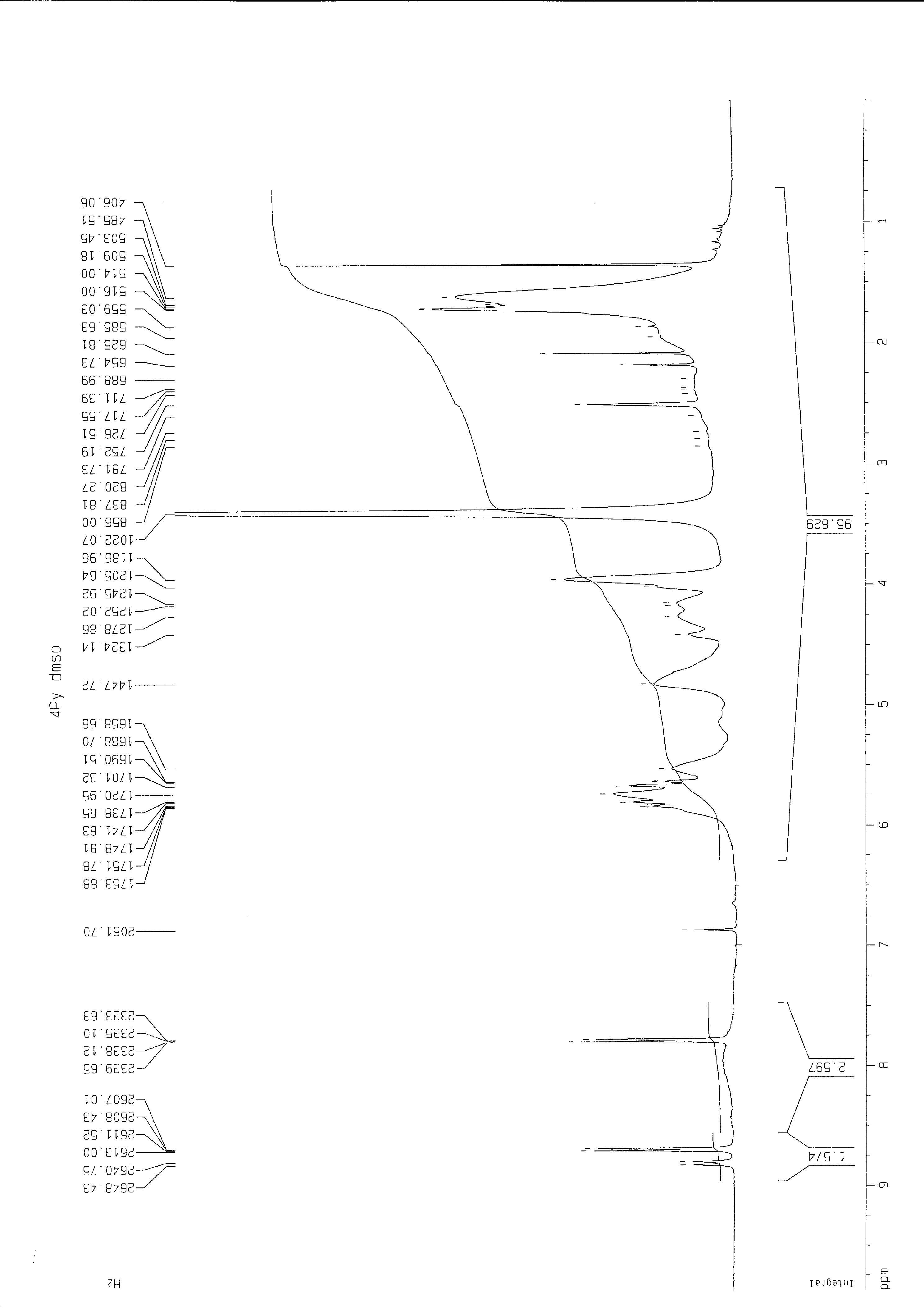
4c


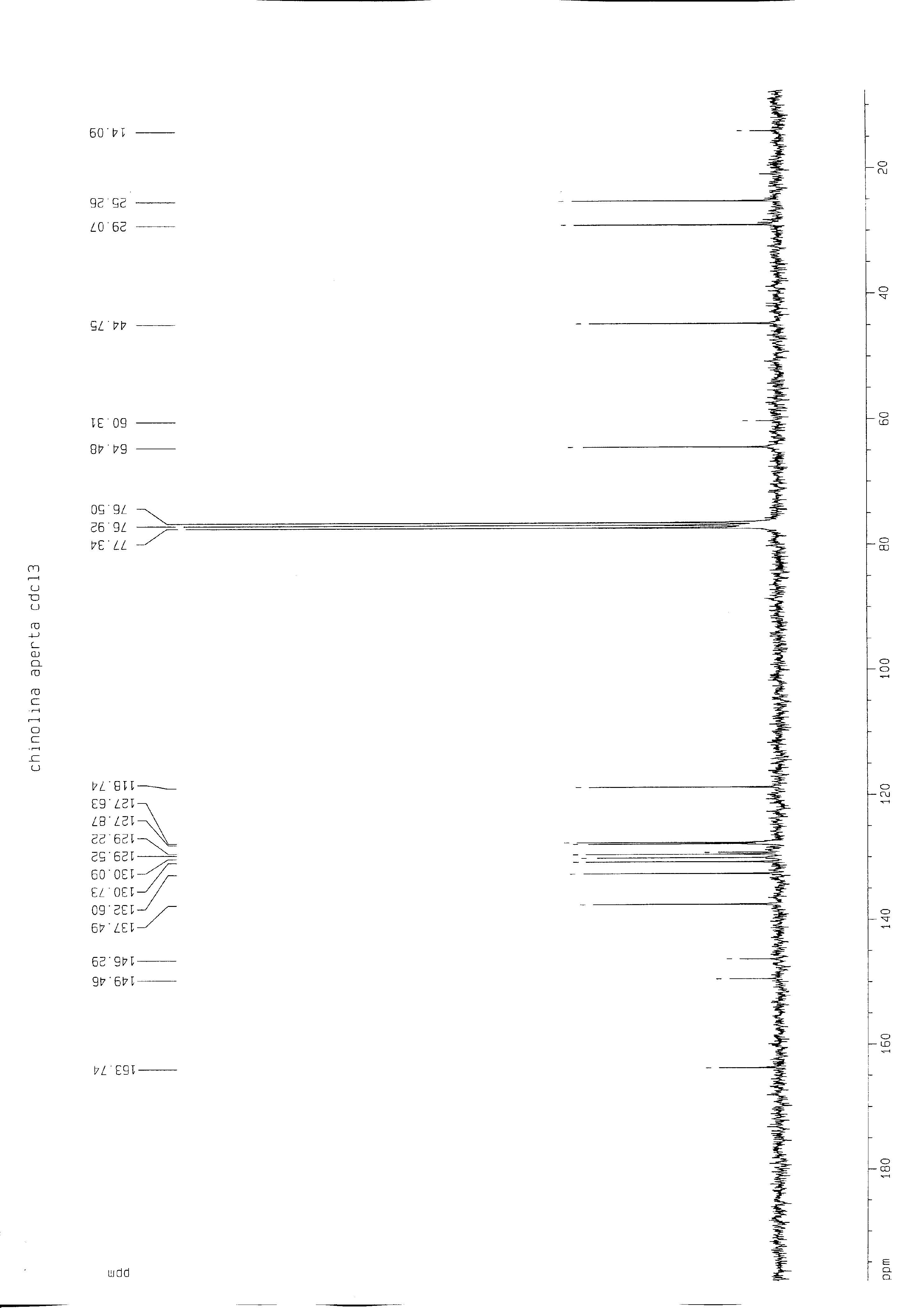
1
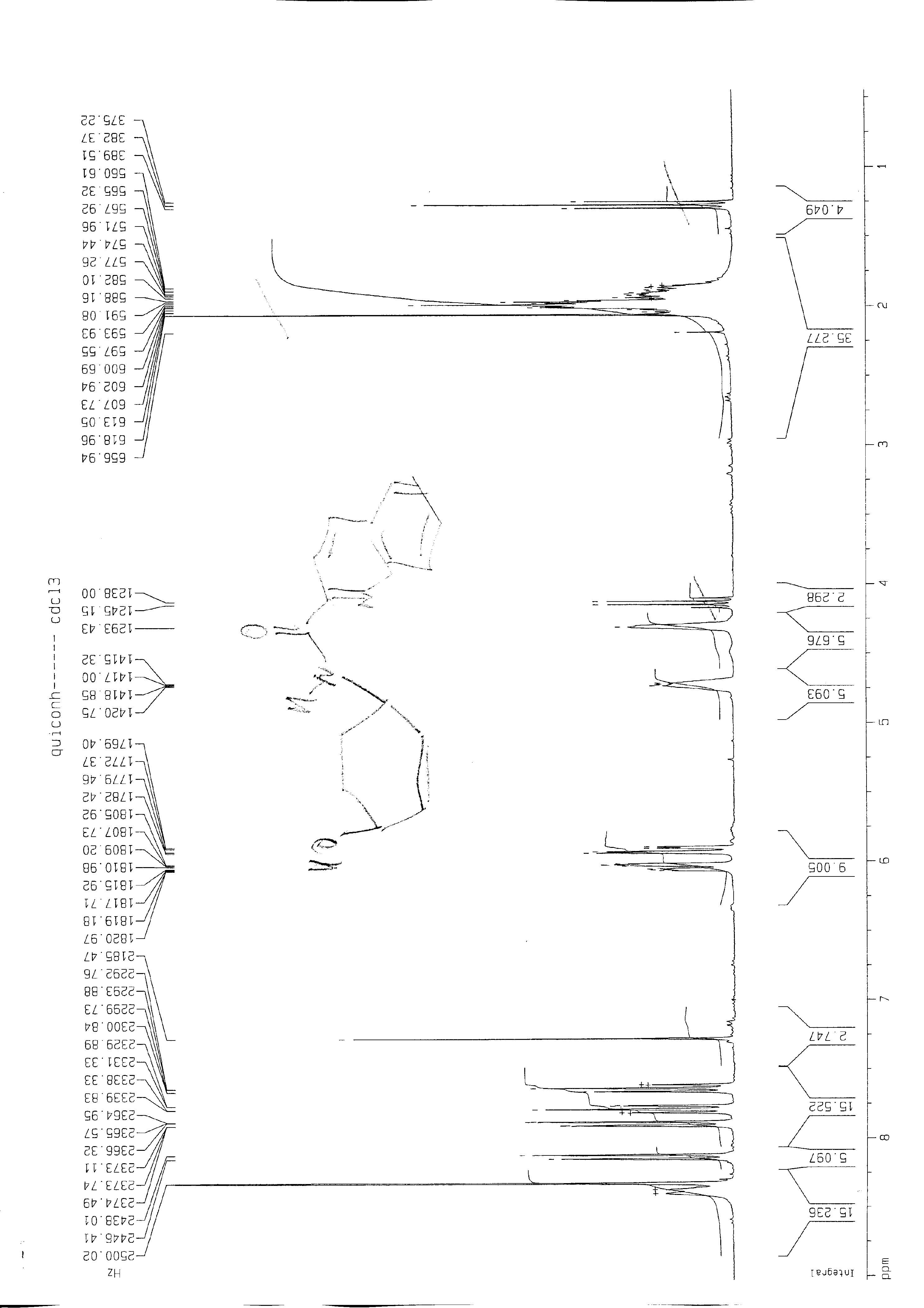
4d


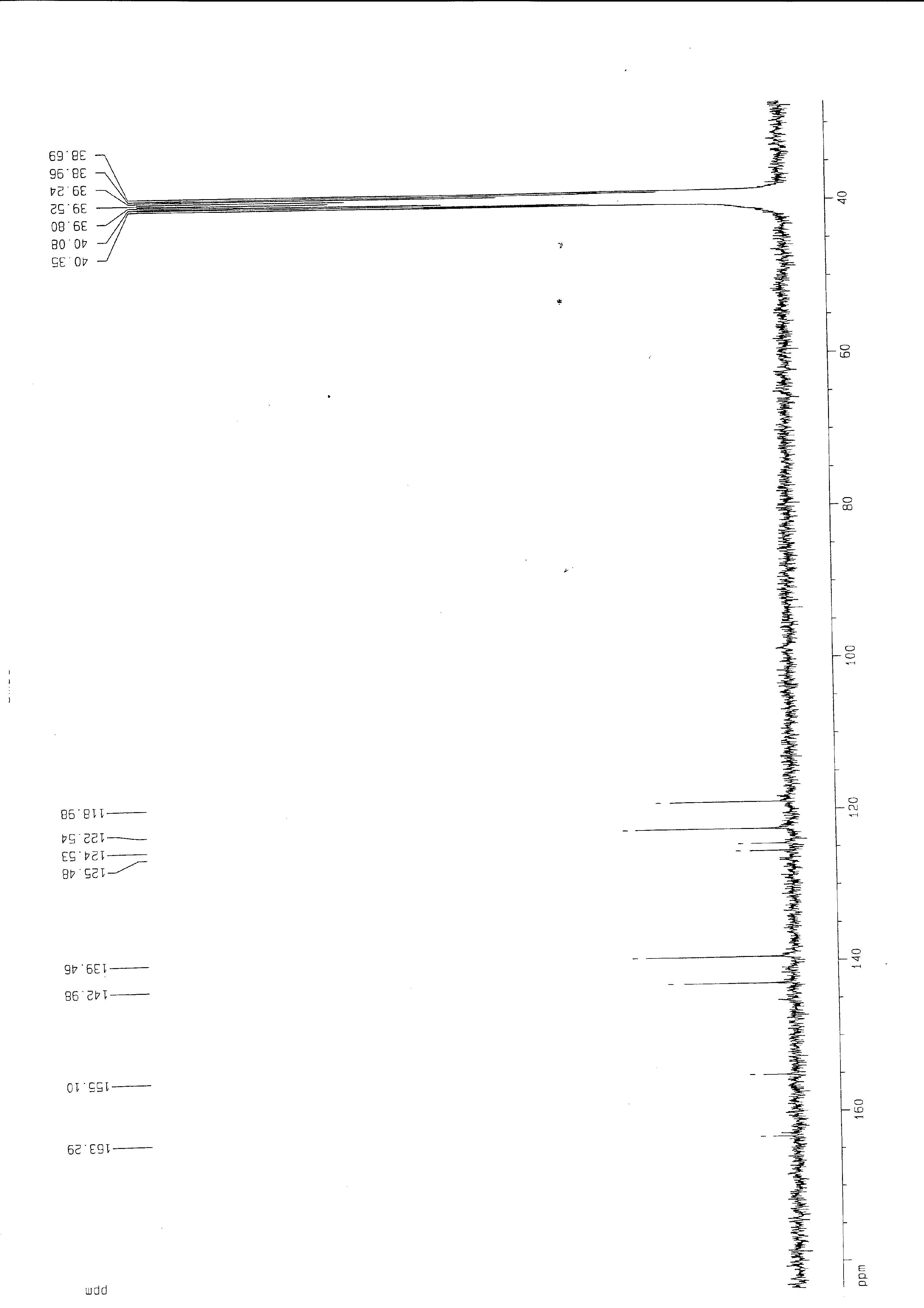


2. Cartesian coordinates of calculated structures

**14d**

1

C -2.63917600 -1.13860700 0.16986500

C -3.46115200 -0.85440000 -1.10309900

C -4.09896700 0.53793200 -1.05478700

H -4.24790900 -1.61618000 -1.18310000

H -2.81572700 -0.97203600 -1.97790200

C -5.06381800 0.64133900 0.13196400

H -4.62555500 0.75974000 -1.99135200

H -3.32562600 1.30576200 -0.92899900

C -4.43231200 0.11554500 1.39525800

C -3.36434900 -0.68707300 1.41614400

H -4.91629300 0.41930000 2.32071900

H -2.95950500 -1.03042100 2.36615000

H -5.95714100 0.02567500 -0.08304200

O -5.46588000 1.98724300 0.39727900

H -5.95362600 2.30115800 -0.37567700

H -2.43409000 -2.21347700 0.21926800

N -1.31949700 -0.49189400 0.13327000

H -1.20247700 0.37418500 0.63812300

O -0.25971500 -2.27396000 -0.81377500

C 1.07795100 -0.35077800 -0.26074000

C 1.09231000 1.04694400 -0.53130400

C 3.34940600 -0.38314700 0.11160700

C 2.28676400 1.72399200 -0.47339800

H 0.17580600 1.55668400 -0.80943900

C 4.51362900 -1.12632100 0.44589400

C 3.47082800 1.02229200 -0.13917900

H 2.33637800 2.78856400 -0.68781700

C 5.73735500 -0.50262700 0.52533200

H 4.39528300 -2.18888700 0.62937800

C 4.74773200 1.63584300 -0.04964800

C 5.85640100 0.88848000 0.27625800

H 6.62365000 -1.07625900 0.77967700

H 4.83477500 2.70207600 -0.24197300

H 6.83091500 1.36272700 0.34373100

C -0.21265400 -1.14647900 -0.34357700

N 2.16038700 -1.04274800 0.04025700

2

C -2.60989800 -1.00602800 0.54224900

C -3.36241000 -1.04227700 -0.80471900

C -3.70532400 0.36930100 -1.29199400

H -4.28768800 -1.61756800 -0.66728600

H -2.75720300 -1.58584300 -1.53532900

C -4.62542700 1.06918900 -0.28557100

H -4.18161500 0.33576800 -2.27997600

H -2.79346700 0.97045500 -1.39284000

C -4.10049300 0.92759600 1.11986100

C -3.21503100 0.00028100 1.49426400

H -4.50235500 1.63463800 1.84210100

H -2.87342500 -0.04188600 2.52647100

H -5.62301600 0.59285500 -0.32691200

O -4.75651400 2.46949600 -0.54104600

H -5.15734800 2.56728100 -1.41493600

H -2.64671300 -2.00870400 0.98283400

N -1.18647200 -0.70315300 0.38186700

H -0.84621500 0.24805700 0.44688000

O -0.52025500 -2.86483600 0.04198600

C 1.16001900 -1.15589600 0.04210600

C 2.19229300 -2.09794500 -0.19745000

C 2.63888000 0.61253100 0.08224700

C 3.48366400 -1.64288100 -0.29542300

H 1.92396500 -3.14292900 -0.29356300

C 2.86715700 2.00648700 0.22764400

C 3.75254200 -0.25641800 -0.15638300

H 4.30656500 -2.32932700 -0.47798300

C 4.14320000 2.51432700 0.14058700

H 2.00821700 2.64437400 0.40823300

C 5.05515600 0.30004200 -0.24120900

C 5.24595300 1.65564900 -0.09560400

H 4.31165900 3.58111400 0.25338200

H 5.89809900 -0.36161200 -0.42231800

H 6.24580700 2.07394100 -0.16125500

C -0.26119000 -1.66672200 0.15249400

N 1.36280600 0.14296600 0.17618700

3

C -0.14335000 2.25108100 -0.30295100

O -0.02384000 3.43843100 -0.60794200

N -1.38065600 1.69892700 -0.14409900

H -2.10958800 2.36786900 -0.36506200

C -1.83441800 0.36897100 0.27702000

C -2.25434600 -0.49607800 -0.92214400

H -0.98782300 -0.11131900 0.77426900

C -2.99449400 -1.74713700 -0.44071600

H -2.92478500 0.08638800 -1.56480100

H -1.36415300 -0.75005900 -1.50404100

C -4.28969900 -1.37249500 0.29641800

H -3.23288600 -2.40861100 -1.28303800

H -2.35350400 -2.32476800 0.23957100

H -4.65050400 -2.24977300 0.85847600

C -4.06312500 -0.24305100 1.27076200

C -2.97053600 0.52493800 1.26481800

O -5.31582100 -0.93308700 -0.60677000

H -5.59273000 -1.70111200 -1.12497400

H -4.85610400 -0.05775600 1.99134800

H -2.85628400 1.31715900 2.00298700

C 1.11228000 1.42420500 -0.10955300

C 2.31323400 2.15587500 0.10748300

C 2.23823200 -0.59109900 -0.08526700

C 3.49196600 1.46868300 0.24331300

H 2.25629900 3.23616200 0.14751800

C 2.19780400 -2.00609500 -0.19555100

C 3.49451800 0.05283700 0.14809000

H 4.42779800 1.99374400 0.41706000

C 3.35161000 -2.74702200 -0.07496000

H 1.23523800 -2.47339600 -0.37574900

C 4.66571100 -0.73862700 0.26744000

C 4.59531900 -2.10948000 0.15853900

H 3.31356500 -3.82899900 -0.15911500

H 5.61763600 -0.24501400 0.44454500

H 5.49580800 -2.70921100 0.25064300

N 1.07138000 0.10720400 -0.20567100
